# Evidence on the Relationships Between Total Fat Intake and Health Outcomes Other Than Unhealthy Weight Gain in Adults and Children: A Scoping Review

**DOI:** 10.1002/cesm.70104

**Published:** 2026-08-28

**Authors:** Marianne Visser, Sarah Gordon, Amanda Brand, Anel Schoonees, Celeste Naude

**Affiliations:** ^1^ Centre for Evidence‐based Health Care, Division of Epidemiology and Biostatistics, Department of Global Health, Faculty of Medicine and Health Sciences Stellenbosch University Cape Town South Africa

**Keywords:** dietary intake, health outcomes, scoping review, systematic review, total fat intake

## Abstract

**Introduction:**

Global guidance provides evidence‐informed recommendations on total fat intake to prevent unhealthy weight gain in adults and children, however, evidence regarding total fat intake and other health outcomes, particularly non‐communicable diseases (NCDs), also exists. This scoping review aimed to map and identify gaps in this body of evidence.

**Methods:**

Four databases were searched from 2014 to 2024 for published systematic reviews (SRs), in any population, assessing the effects or associations of reduced total fat intake (e.g., ≤ 30% total energy [TE]), compared with usual fat intake (e.g., > 30%TE), on pre‐specified health outcomes other than measures of unhealthy weight gain or acute post‐prandial outcomes. We screened independently and in duplicate. Data were extracted, checked, and narratively summarized inductively by health topic.

**Results:**

Sixty‐seven SRs were included. Eight were *fully* aligned (all included studies addressed the scoping review question) and reported on health topics related to NCDs, for example, cancers (gastrointestinal, hematological), bone and joint health, gastrointestinal and neurological disorders, and cardiometabolic outcomes. Fifty‐nine SRs *partially* addressed the scoping review question, reporting additional topics, for example, cancers (breast, gynecological, urological, liver, skin), and respiratory and liver disorders. Almost one third of included SRs (*n* = 21) reported outcomes related to cardiometabolic risk and cardiovascular disease, obtained mostly from randomized controlled trial (RCT) evidence. Evidence for other topics was mostly observational. Overall, evidence was predominantly from high‐income countries, and in adults.

**Conclusions:**

This scoping review maps available evidence on the relationships between total fat intake and health outcomes other than unhealthy weight gain and acute postprandial effects and demonstrated a heterogenous body of evidence, with several evidence gaps, predominantly relating to NCDs.

## Plain Language Summary

Global guidance on the total amount of fat to be consumed in the diet to prevent unhealthy weight gain does not focus on other health measures. This scoping review mapped all the health evidence on the relationship between total dietary fat intake as a percentage of daily energy intake and measures other than unhealthy weight gain.

We searched four electronic databases for systematic reviews, published from 2014 to 2024. Systematic reviews needed to include studies comparing reduced total fat intake with typical fat intake, in any population. Two authors of our scoping review decided whether systematic reviews should be included, whereafter information was summarized by health topic.

Sixty‐seven systematic reviews were included. In eight systematic reviews all the included studies answered our scoping review question (*fully* aligned). These reported on health topics, for example, cancer, bone and joint health, digestive tract and nervous system disorders, and heart and metabolic health. Fifty‐nine systematic reviews included some studies answering our scoping review question (*partially* aligned). These reported additional health topics, for example, other cancers, and airway and liver disorders. Almost a third of included systematic reviews reported outcomes related to heart and metabolic health, mostly from clinical trials. Evidence for other topics was mostly from studies that observe people over time. Most evidence was from high‐income countries, and in adults.

The findings from this scoping review may inform the development of future guidance on total dietary fat intake of adults and children in relation to a variety of health measures, particularly those related to lifestyle diseases.

## 1 Introduction

Total dietary fat intake closely correlates with energy intake, which influences body weight and body fatness [1, 2]. Anthropometric measures such as body weight, body mass index, waist circumference, skinfold thickness and percentage body fat are commonly used as outcomes to assess weight change, including unhealthy weight gain. In this context, unhealthy weight gain refers to unintended weight gain (i.e., body fatness increase) that contributes to the progression towards overweight and obesity. It excludes appropriate weight gain during pregnancy, as part of normal childhood growth and development, and weight gain resulting from activities that increase muscle mass without increasing fat mass, such as weight‐lifting and other strength‐building activities [3]. A 2023 World Health Organization (WHO) guideline provided evidence‐informed recommendations on total fat intake for the prevention of unhealthy weight gain in adults and children. This guideline is part of WHO's ongoing work towards updating dietary recommendations to reduce the global burden of obesity and related health conditions [3].

The relationships between total fat intake and many other health outcomes and conditions, including certain cancers, cardiovascular disease and type 2 diabetes mellitus, have also been investigated in observational and experimental studies [4, 5, 6]. This scoping review was conducted to explore and better understand the evidence base on the relationships between total fat intake and health outcomes other than unhealthy weight gain, to inform the scope of the WHO NUGAG subgroup on Diet and Health's ongoing work on dietary recommendations at the time. Scoping reviews are a type of knowledge synthesis that follow systematic methods to map the breadth of evidence available on a topic, field or concept within and across particular contexts [7]. A comprehensive mapping of the depth and breadth of evidence on relationships between total fat intake and health outcomes other than measures of unhealthy weight gain, may be useful to inform new research, in addition to updating current recommendations on total fat intake of adults and children.

The objectives of this scoping review were to:

1. Identify and map existing evidence on the relationships between total fat intake and health outcomes, other than measures of unhealthy body weight gain, using a systematic approach based on pre‐defined eligibility criteria, and
2. Identify gaps in the evidence base.

This review will complement the completed evidence syntheses on the effects of total fat intake on body fatness in children and adults [2, 8].

## 2 Methods

Methods were informed by guidance for scoping reviews [9] and reporting followed a systematic approach, informed by the PRISMA Extension for Scoping Reviews (PRISMA‐ScR) reporting guideline [10].

### 2.1 Criteria for Considering Studies for This Review

Published systematic reviews (SRs) meeting the eligibility criteria outlined in Table 1 were included. In brief, SRs of randomized controlled trials (RCTs) assessing interventions aiming to reduce total fat intake (e.g., 30% or less of total energy [TE]) compared with usual/higher fat intake (e.g., greater than 30% TE) were eligible, regardless of the indication for adjustments to total fat intake. Any SR reporting the effects of a reduced total fat intake (e.g., low‐fat diets) without the inclusion of an eligible comparator, was excluded. SRs of cohort and case‐control studies comparatively analyzing total fat intake exposure (i.e., lower fat intake vs. usual or modified fat intakes) were eligible if they set out to examine the effects or associations of total fat intake in relation to any health outcomes (e.g., conditions, symptoms, or risk factors) other than measures of unhealthy weight gain. SRs in children or adults of any age and from any setting, published in English, were eligible. SRs of cross‐sectional studies and qualitative reviews, as well as SRs lacking comparative analysis or focusing solely on fat quality, were excluded.

**TABLE 1:** Eligibility criteria for this scoping review.

Item	Eligibility criteria
**Population**	Children (from birth) and adults from any country or setting.
**Comparisons of interest**	Interventions stating an intention to reduce total fat intake (e.g., 30% or less of TE intake) by provision of foods, nutrition education in any form, or both, compared with usual total fat intake (e.g., greater than 30% TE from fat) or modified fat intake (e.g., greater than 30% TE from fat and higher levels of monounsaturated or polyunsaturated fats than a “usual“ fat diet). Total fat intake exposure (in grams, as a percentage of TE intake, or as one of the defining characteristics of a diet or dietary pattern) comparatively analyzed (e.g., lower vs. higher intakes) in relation to eligible outcome measures over time (prospectively or retrospectively). We excluded: − reviews *only* examining different types of dietary fat (i.e., quality of fat) without also including total fat intake; − reviews examining cross‐sectional associations with eligible outcomes; and − reviews with no comparative analysis, that is, only descriptive results with no analyses examining associations between different levels of total fat intake and eligible outcomes.
**Outcomes**	All health outcomes, particularly related to non‐communicable diseases, such as certain cancers, cardiovascular disease and type 2 diabetes mellitus. Reviews were included *only* if the *aim(s)* and/or *primary* outcomes of the review were focused on conditions/symptoms/risk factors *other than* measures of unhealthy weight gain (e.g., carcinoma, mental health, cardiovascular health). Reviews were excluded if the aim(s) and/or primary outcomes were focused on measures of unhealthy weight gain or body fatness, or acute and post‐prandial outcomes (e.g., dyspeptic symptoms).
**Study designs and language**	SRs of RCTs, cohort and case‐control studies assessing effects or associations of total fat intake on eligible health outcomes, in English, were included. A SR was defined as a review that had predetermined objectives, predetermined criteria for eligibility, searched at least two data sources, of which one needed to be an electronic database, and performed data extraction of included studies. SRs that did not align with this definition were excluded. SRs of qualitative or cross‐sectional studies were excluded. SRs that also included studies with designs other than RCTs and/or cohort and/or case‐control studies were included *only* if it was possible to extract the required data separately from the studies with eligible designs (i.e., RCTs, cohort, case‐control studies).

Abbreviations: RCT, randomized controlled trials; SR, systematic review; TE, total energy.

### 2.2 Search Methods for the Identification of Studies

In July 2020 the following four databases were searched: MEDLINE (via PubMed), Cochrane Database of Systematic Reviews (CDSR), Epistemonikos and Turning Research into Practice (TRIP), using a comprehensive search strategy, to identify eligible SRs published in 2014–2020. In January 2024, the search was updated. The search strategy was developed and implemented with support from a Cochrane information specialist. The search strings per database are detailed in Supporting Information S1: Table 1.

## 3 Data Collection and Analysis

### 3.1 Study Selection

The titles and abstracts of records identified through the searches were screened against the eligibility criteria using the Rayyan QCRI web application [11]. Potentially eligible full texts were then obtained and screened for eligibility. All screening and selection were done independently in duplicate. For all screening, if it was clear that the primary outcomes were focused on measures of unhealthy weight gain or body fatness, or acute and post‐prandial outcomes, the SR would be excluded. The selection process was recorded by completing a PRISMA flow diagram [12]. A single reviewer extracted relevant data from all included SRs using a piloted data charting form. All data extraction from the initial search yield was checked by a second reviewer for quality control and 20% of the data extraction for the updated search was checked for consistency.

### 3.2 Data Extraction and Charting

Data were extracted for SRs where all the included primary studies only evaluated the relationship between total fat intake and health outcomes meeting our inclusion criteria and did not evaluate any other interventions or exposures (*fully* aligned SRs). Data were extracted separately for those SRs where only a portion of included primary studies examined total fat intake in relation to eligible health outcomes (*partially* aligned SRs). In these SRs, exposures often went beyond total dietary fat intake (e.g., intakes of different types of fatty acids, macro‐ or micronutrients, foods or food groups, and non‐dietary exposures such as physical activity).

Comprehensive data charting of *fully* aligned SRs captured details on: first author, publication date, number of primary studies included in each SR that examined total fat intake, study designs of included primary studies and countries where these studies were conducted, details of the PICO elements of included SRs from the methods and results sections, and research gaps described by authors of the included SRs (using the Evidence, population, intervention, comparison, outcome, time stamp, study design, time frame and burden of disease (EPICOT + ) framework for formulating research recommendations) [13].

Data charting for *partially* aligned SRs used a streamlined version of the piloted data charting form, capturing details on first author, publication date, number of primary studies included in each SR that examined total fat intake, study designs of included primary studies, details of the PICO elements of included SRs from the methods. Data were not extracted from the results section of *partially* aligned SRs as these findings would not be fully applicable to this scoping review, given their partially aligned nature.

Both *fully* and *partially* aligned included SRs were categorized according to broad health topics, informed by pre‐specified outcomes reported in study populations, as defined by each SR. The methodological quality of included SRs was not assessed since this review aimed to *scope* the evidence base.

## 4 Results

### 4.1 Results of the Search

**FIGURE 1:**
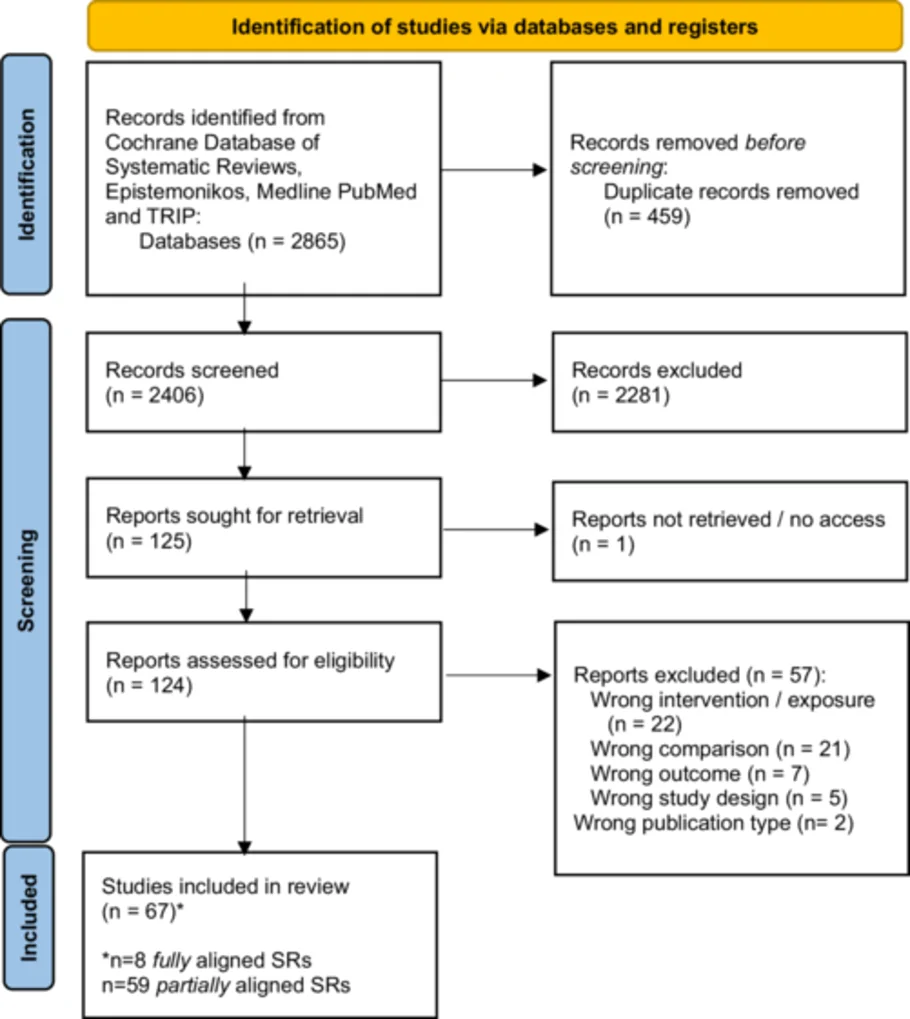
Study selection flowchart [12].

The study selection flowchart is available in Figure 1. After screening the titles and abstracts of 2406 de‐duplicated records, we assessed the full texts of 124 records against our eligibility criteria and included 67 SRs (*fully* aligned n = 8, *partially* aligned *n* = 59) after excluding 56 full‐text records [2, 14, 15, 16, 17, 18, 19, 20, 21, 22, 23, 24, 25, 26, 27, 28, 29, 30, 31, 32, 33, 34, 35, 36, 37, 38, 39, 40, 41, 42, 43, 44, 45, 46, 47, 48, 49, 50, 51, 52, 53, 54, 55, 56, 57, 58, 59, 60, 61, 62, 63, 64, 65, 66, 67, 68] and one duplicate [51]. The reasons for exclusion of each of these records are summarized in Supporting Information S1: Table 2.

### 4.2 Included Studies

The characteristics of all the included SRs (*n* = 67) are summarized in Supporting Information S1: Tables 3 and 4. In eight of the 67 SRs, all included primary studies reported on total fat intake and eligible health outcomes and were therefore classified as *fully* aligned SRs (add refs) (Supporting Information S1: Table 3). The remaining 59 were classified as *partially* aligned (Supporting Information S1: Table 4).

SRs were *partially* aligned if they also assessed exposures other than total dietary fat intake. Many SRs examined a range of additional dietary exposures, for example, the consumption of specific foods or food groups, or different intakes of macro‐ and micronutrients [69, 70, 71, 72, 73, 74, 75, 76, 77, 78, 79, 80, 81, 82, 83, 84, 85, 86, 87, 88]. Several *partially* aligned SRs assessed the relationship of different types of dietary fatty acids (i.e., saturated, mono‐ and polyunsaturated fatty acids) [5, 6, 89, 90, 91, 92, 93, 94, 95, 96, 97, 98, 99, 100], or biomarkers of fatty acids [101], with eligible health outcomes. Some SRs were *partially aligned* as they assessed lifestyle exposures or interventions, such as physical activity, in addition to dietary exposures or interventions [88, 102, 103].

### 4.3 Outcomes Addressed by *Fully* Aligned SRs

Eight *fully* aligned SRs (Supporting Table S3) addressed the relationship between total fat intake and a wide range of health outcomes. We grouped these SRs according to seven broad health topics (see Box 1). Box 1 provides a summary of all the pre‐specified health outcomes meeting our inclusion criteria, reported by SR authors.

Box 1. **Summary of pre‐specified outcomes reported by *n* = 8 included *fully* aligned SRs (categorized by health topic).**

**Table:** 

Cancer – gastrointestinal (*n* = 1)	Cancer – hematological (*n* = 1)	Bone & joint (*n* = 1)	Endocrine (*n* = 1)
Pancreatic cancer risk	Non‐Hodgkin's lymphoma risk	Fracture risk	Testosterone Sex hormone binding globulin Luteinising hormone

Gastrointestinal (*n* = 1)	Cardiometabolic (*n* = 2)		Cognitive and Neurological (*n* = 1)
Ulcerative colitis risk Crohn's disease risk	DBP SBP LDL‐C HDL‐C TC TAG	Insulin Glucose Leptin Adiponectin Hs‐CRP	Dementia risk Alzheimer's disease risk

**Abbreviations:** CVD, cardiovascular disease; DBP, Diastolic blood pressure; HDL‐C, High‐density lipoprotein cholesterol; Hs‐CRP, High sensitivity C‐reactive protein; LDL‐C, Low density lipoprotein cholesterol; SBP, Systolic blood pressure; TAG, triacylglycerol; TC, Total cholesterol.

Most *fully* aligned SRs included only observational studies, where analyses compared the highest category of total fat intake with the lowest category of total fat intake. The exception was for the Cardiometabolic topic, where SRs included only RCTs comparing lower‐fat diets with higher‐fat diets. In the section below, the main characteristics of the included *fully* aligned SRs, as well as the research gaps identified by the authors of these included SRs, is summarized according to each health topic.

### 4.4 Main Characteristics of Included *Fully* Aligned SRs and Research Gaps Identified

#### 4.4.1 Cancer—Gastrointestinal

One SR published in 2015 assessed total fat intake in relation to pancreatic cancer [104]. Shen and colleagues assessed associations between the intake of total fat and *pancreatic cancer risk* in adults, (i.e., either men only, women only, or both), reported by six cohort studies (*n* ≈ 1,064,123) that followed study participants from eight to 22 years, and 13 case‐control studies (*n* ≈ 10,502). The highest categories of total fat intake were compared to the lowest categories of intake. Included studies were conducted in high‐ and upper‐ middle income economies in North America (Canada, USA), Europe (Finland, Greece, Italy, Netherlands, Poland), Oceania (Australia) and Asia (Japan) and in Asia (China). Research recommendations by SR authors outlined that, in terms of evidence, prospectively designed studies or pooled studies of a large consortium are needed to perform a dose‐response analysis between total fat intake and pancreatic cancer risk. In addition, SR authors recommended that future studies should adjust for TE intake and consider adjustment for the consumption of red and/or processed meat, since this variable has been associated with an increased pancreatic cancer risk [104].

#### 4.4.2 Cancer—Hematological

One SR published in 2017 examined the association between total fat consumption and *non‐Hodgkin's lymphoma (NHL)*, as reported by two cohort studies (*n* ≈ 123,566; follow‐up not reported) and eight case‐control studies (*n* ≈ 13,057) conducted in adults, that is, either women only, men only, or both [105]. During analyses the highest category of total fat intake was compared to lowest category of total fat intake. The included studies were all conducted in high‐income economies in North America (Canada, USA), Europe (Italy, Sweden) and Asia (Oman). Research recommendations from this SR included that long‐term prospective studies should be conducted in future to better understand the link between total fat and the *risk of NHL*. Additionally, SR authors stated that such studies be carried out in diverse geographic regions including low‐ and middle‐income countries, to enhance the generalizability of findings. Given that accurate estimates of fat consumption depend on factors such as processing methods, fat source (animal or plant), and measurement tools, SR authors concluded that future research should incorporate standardized dietary assessment methods, including validated questionnaires and advanced techniques for measuring total fat intake.

#### 4.4.3 Bone and Joint

One SR published in 2018 examined the association between total fat intake and *fracture risk* in adults [106]. Four prospective cohort studies (*n* ≈ 172,657) with a mean duration of follow‐up of study participants of 7.6–8.9 years, and three case‐control studies (*n* ≈ 2766), were included. When examining total fat intake, the highest category of total fat intake was compared to lowest category of total fat intake. All included studies were conducted in high‐ and upper‐middle income economies/regions in North America (USA) and Europe (Spain, Sweden) and Asia (China) The research gaps in the evidence base identified by SR authors suggest that more well‐designed studies, including a large number of populations considering both genders and different types of fat, are needed in order to enhance the statistical power of the result examining the association between total fat intake and fracture risk.

#### 4.4.4 Cardiometabolic

Two SRs published in 2014 and in 2018 examined total fat intake and cardiometabolic markers of non‐communicable disease (NCD) risk, specifically *systolic and diastolic blood pressure (SBP and DBP)*, blood lipids comprising *total cholesterol (TC), high*‐ and *low‐density lipoprotein cholesterol (HDL‐C and LDL‐C), triacylglycerol (TAG)*; inflammatory markers (*high‐sensitivity C‐reactive protein, Hs‐CRP*) and markers of glucose and fat metabolism (including *leptin* and *adiponectin)* in adults [107, 108]. The 2018 review included 20 RCTs (*n* ≈ 2106) conducted in metabolically healthy overweight adults, with a range of follow‐up of study participants of between 6 weeks and 2 years [107]. The 2014 review included 14 RCTs (*n* ≈ 1753) conducted in adults with a diagnosis of pre‐diabetes mellitus or type 2 diabetes mellitus (T2DM), with a duration of follow‐up of study participants of at least 12 months, up to 6 years [108]. When analyzing total fat intake, Lu et al. compared the effects of low‐fat diets (< 30% of TE) to high‐fat diets (> 30% of TE), excluding studies where high‐fat diets were defined as only 30% of TE [107], while Schwingshackl and colleagues used a similar approach by comparing high fat intakes (> 30% of TE) to low fat intakes (≤ 30% of TE) [108]. One SR did not report on the economies of origin of their included studies [108], while all included studies in the other SR were conducted in high‐income economies/regions in North America (Canada, USA), Europe (Denmark, Germany, Spain, Sweden), and Oceania (Australia) [107]. Authors of both SRs suggested that more long‐term high‐quality RCTs are warranted using a standardized approach to elucidate the benefits and disadvantages of both low‐fat and high‐fat dietary regimes, including the additional effects of different fat types on cardiometabolic outcomes.

#### 4.4.5 Cognitive and Neurological

One SR examining the associations of total fat intake in relation to *dementia* and *Alzheimer's disease risk* included four prospective cohort studies that followed 8630 healthy adults between 2.1 years and 21 years [109]. When analyzing total fat intake, the review compared highest categories of total fat intake to lowest categories in relation to their outcomes. The SR included studies conducted in high‐income economies in North America (USA), and Europe (Finland, Netherlands). To enhance the evidence base on the association between total fat intake and cognitive and neurological outcomes, SR authors identified the need for additional prospective studies.

#### 4.4.6 Gastrointestinal

One SR published in 2016 examined total fat intake and risk of inflammatory bowel disease *(ulcerative colitis and Crohn's disease risk)* and included three cohort studies (*n* ≈ 395,848; follow‐up not reported) and five case‐control studies (*n* ≈ 2233) studies conducted in participants aged 14 years and older [110]. When analyzing total fat intake, the review compared the highest categories of total fat intake to the lowest categories. All included studies were conducted in high‐income economies in North America (Canada, USA), Europe (France, Japan, Netherlands, Sweden) and Asia (Japan, Israel). SR authors suggested that more prospective studies are needed, especially in countries other than Asia, in order to strengthen the findings from their review.

#### 4.4.7 Endocrine

One SR published in 2021 examined total fat intake in relation to *testosterone* and *related hormone markers* in healthy adult men [111]. Six trials (*n* ≈ 206), both randomized and non‐randomized, with durations between two and 10 weeks, and a difference in total fat intake between treatment groups of at least 10%TE, were included. When examining total fat intake, Whittaker et al. compared the effects of low‐fat diets to high‐fat diets. The included studies were mostly conducted in high‐income economies in North America (USA) and Europe (Finland), whereas one study included two groups of study participants from both North America (USA) and South Africa, respectively. Gaps in the research base were reported by SR authors as the need for large RCTs to confirm the findings from their review before any practical recommendations can be made. In addition, it was suggested that future studies should also investigate the effects of ethnicity on androgen metabolism, in response to dietary fat intake.

### 4.5 Outcomes Addressed by *Partially* Aligned SRs

Fifty‐nine included SRs partially addressed the relationships between total fat intake and a wide range of health outcomes (Supporting Information S1: Table 4). We grouped these *partially* aligned included SRs according to 12 broad health topics (see Box 2). Only a proportion (ranging from 1.1% to 95.5%) of the total number of included primary studies in these SRs reported on total fat intake in relation to some of the prespecified SR outcomes within each health topic. In the section below the characteristics of the included *partially* aligned SRs is summarized according to each health topic.

Box 2. **Summary of pre‐specified outcomes reported by 59 included *partially* aligned SRs (categorized by health topic).**

**Table:** 

Cancer – breast (*n* = 6)	Cancer – gastrointestinal (*n* = 4)		Cancer – gynecological (*n* = 6)	Cancer – urological (*n* = 2)
Breast cancer risk Mortality (all cause, breast cancer specific, CVD) Breast cancer recurrence/progression Other primary cancer risk Survival QoL	Gastric cancer risk Esophageal adenocarcinoma risk Esophageal squamous cell carcinoma risk Colorectal cancer risk Mortality Morbidity		Ovarian cancer risk Epithelial ovarian cancer risk Endometrial cancer risk	Bladder cancer risk Prostate cancer risk

Cancer – skin (*n* = 1)	Cancer – overall (*n* = 1)	Liver (*n* = 4)	Cognitive and Neurological (*n* = 8)	
Skin cancer risk (Basal cell carcinoma risk, Squamous cell carcinoma risk, Cutaneous malignant melanoma risk)	Survival (Overall, Disease free, Recurrence free) Mortality (All‐cause; Cancer‐specific; cause other than specific cancer) Cancer progression/recurrence	Liver fat content Liver enzymes Liver echogenicity Glucose Insulin TC TG HDL‐C LDL‐C	Cognitive function Mild cognitive impairment risk Dementia risk Alzheimer's disease risk Parkinson's disease risk Pain Fatigue QoL	

Cardiometabolic and Cardiovascular (*n* = 19)				
Mortality (all‐cause, CVD, CHD, stroke, T2DM and cancer‐specific)	CVD risk (CHD, stroke, MI, angina, atrial fibrillation, heart failure, peripheral arterial disease)	Inflammatory markers Arterial stiffness T2DM and gestational diabetes risk DM‐associated morbidity	QoL Adiponectin Insulin Glucose HbA1c MetS BP/HT	LDL‐C/ox‐LDL VLDL‐C HDL‐C TC TAG/TG ApoB, ApoA‐1 Lp (a)

Cancer – liver (*n* = 1)	Gastrointestinal (*n* = 4)	Endocrine (*n* = 1)	Pregnancy (*n* = 1)	Respiratory (*n* = 1)
Liver cancer risk	Ulcerative colitis risk Crohn's disease risk Gastroesophageal reflux disease risk	Onset of natural menopause	Oxidative stress biomarkers	T2 inflammatory markers

Abbreviations: BP, blood pressure; CHD, coronary heart disease; CVD, cardiovascular disease; HbA1c, glycosylated hemoglobin; HDL‐C, High‐density lipoprotein cholesterol; HT, Hypertension; LDL‐C, Low density lipoprotein cholesterol; Lp (a), Lipoprotein (a); MetS, Metabolic Syndrome; MI, Myocardial infarction; QoL, Quality of life; TAG, triacylglycerol; TC, Total cholesterol; T1DM, Type 1 Diabetes Mellitus; T2DM, Type 2 Diabetes Mellitus.

### 4.6 Main Characteristics of Included *Partially* Aligned SRs

#### 4.6.1 Cancer—Breast

Six SRs, published between 2015 and 2023, addressed *breast cancer risk* in healthy women [81, 86, 89, 112], as well as *mortality (all‐cause, cause‐specific), survival* and *breast cancer recurrence* among women with prior breast cancer [69, 112, 113] and its association with total fat intake, with the percentage of relevant included studies ranging from 2.7% to 83.3%. One SR examined *breast cancer risk*, specifically in healthy Chinese women [86]. All SRs compared the highest to the lowest categories of total fat intake in relation to breast cancer risk, with two SRs also examining dose‐response relationships [69, 89].

#### 4.6.2 Cancer—Gastrointestinal

Four SRs, published between 2015 and 2021, partially addressed the association of total fat intake in relation to various *gastrointestinal cancers* (i.e., *gastric cancer, esophageal adenocarcinoma, esophageal squamous cell carcinoma* and *colorectal cancer risk*) [114, 115, 116, 117]. The percentage of relevant included studies ranged from 1.2% to 95%. All SRs compared the highest to lowest categories of total fat intake regarding gastrointestinal cancer outcomes.

#### 4.6.3 Cancer—Gynecological

Six SRs published between 2014 and 2023 examined the relationship between total fat intake and gynecological cancers (i.e., *ovarian cancer, epithelial ovarian cancer*, and *endometrial cancer risk)* in some of their included studies [5, 71, 118, 119, 120, 121]. The percentage of relevant included studies ranged from 8.3% to 76%. These SRs examined various interventions and exposures alongside total fat intake. Five SRs compared the highest versus lowest categories of total fat intake in relation to gynecological cancer outcomes [71, 118, 119, 120, 121], whereas Sadeghi et al. used a linear dose‐response approach (increments of 10 grams/day in total fat intake) [5].

#### 4.6.4 Cancer—Liver

A single included SR was published in 2021 and evaluated the association between total fat intake and *liver cancer risk* [98]. A total of 35.7% of its included studies were relevant for the question of this scoping review. For total fat intake analyses, the authors compared the highest category of total fat intake to the lowest category in relation to the liver cancer outcomes.

#### 4.6.5 Cancer—Skin

One included SR was published in 2020 and evaluated the association between total fat intake and the risk of three types of skin cancer (i.e., *basal cell carcinoma, squamous cell carcinoma and cutaneous malignant melanoma risk*) [93]. This review was partially relevant with 83.3% of its included studies relevant to the question of the scoping review. For total fat intake analyses, the authors compared the highest category of total fat intake to the lowest category in relation to the three types of skin cancer.

#### 4.6.6 Cancer—Urological

Two SRs, published in 2015 and 2019, examined total fat intake and risk of urological cancer (i.e., *bladder cancer risk* and *prostate cancer risk*) with the percentage of relevant included studies between 90% and 92% [97, 122]. Both SRs examined total fat intake as well as other dietary exposures. When analyzing total fat intake and *bladder cancer risk*, Wang and colleagues compared the highest categories of total fat intake to the lowest categories [97]. For total fat intake and *prostate cancer risk*, per unit increases of 28.35 grams/day in total fat intake were compared to no increase by Xu et al. [122].

#### 4.6.7 Cancer—Overall

One SR, published in 2020, partially addressed the association of total fat intake with *survival (overall, disease‐free, recurrence free), mortality (all‐cause, cancer‐specific)* and *cancer recurrence* in people with breast, gastrointestinal, gynecological, lung, and urological cancers in adults [123]. Only one included primary study (1.3%) was relevant to our question and compared the highest category of total fat intake to the lowest category of fat intake.

#### 4.6.8 Cardiometabolic and Cardiovascular

Out of total of 19 SRs in this category, twelve SRs published between 2014 and 2024 examined the relationship between total fat intake and *cardiovascular disease (CVD) risk* [6, 73, 79, 80, 100, 101, 102, 124, 125], *T2DM risk* [6, 70] and *mortality (all‐cause, cause specific, e.g., CVD, T2DM)* [6, 73, 79, 80, 91, 92, 101, 102, 124], with the percentage of relevant included studies ranging from 1.2% to 70%.

Twelve SRs published between 2014 and 2023 examined total fat intake and cardiometabolic markers of NCD risk in healthy (*n* = 7) and at‐risk (*n* = 4) populations. One SR looked at NCD risk biomarkers but did not prespecify outcomes [78], while the other SRs specifically focused on outcomes such as *metabolic syndrome (MetS)* [79, 90], *blood pressure* [73, 79, 125], *blood lipids* [73, 79, 125, 126], *inflammatory markers* [6], *markers of glucose metabolism (including insulin resistance)* [70, 126, 127, 128], *adiponectin* [95], mainly in adults, and *arterial stiffness* in adolescents and children [77]. One SR reported several of these cardiometabolic risk markers, specifically within the Korean population [125]. The percentage of relevant included studies ranged from 1.2% to 47.1%.

When analyzing total fat intake, five SRs compared low total fat with high total fat intake from RCT evidence [95, 102, 124, 127, 128], with some using approaches such as meta‐regression [95]. Six SRs compared higher fat intake with lower fat intake from observational evidence [80, 91, 92, 100, 101, 125], with some specifying dose‐response analyses [80, 100, 101]. Five SRs compared various dietary patterns [70, 73, 78, 79] or diets [126]. Three SRs did not specify their comparison methods [6, 77, 90].

#### 4.6.9 Cognitive and Neurological

Six SRs published between 2015 and 2023 examined total fat intake in relation to cognitive and neurological outcomes in adults; more specifically, changes in *cognitive function* [129], *mild cognitive impairment, dementia* (Cao 2019, Zhu 2021, Townsend 2023), and *risk of Alzheimer's* [99, 130] and *Parkinson's disease* [84, 131] were assessed. In addition, two additional SRs assessed total fat intake in relation to other neurological outcomes such as *neuropathic pain* [132], and general health outcomes such as *fatigue* and *quality of life (QoL)* in a SR conducted in people with multiple sclerosis [82]. The percentage of relevant included studies ranged from 1.1% to 77%. When analyzing total fat intake, two SRs compared highest categories of total fat intake to lowest categories in relation to their outcomes [84, 130], and one SR compared a high total fat intake (i.e., within 4th quartile) to a moderate total fat intake (i.e., 2nd, 3rd quartiles) [131]. In addition, three SRs investigated the effects of low‐fat diets [82], diets with a decreased total fat content [129] and low‐fat dietary patterns [83].

#### 4.6.10 Gastrointestinal

Four SRs examined total fat intake and gastrointestinal outcomes (i.e., *ulcerative colitis risk* [96], *Crohn's disease risk* [87] and *gastroesophageal reflux disease* [76, 88]. The percentage of relevant included studies ranged from 1.4% to 67%. When analyzing total fat intake, two SRs compared the highest categories of total fat intake to the lowest categories [87, 88], while a third one compared per unit increases of 30 grams/day in total fat to no increase [96]. Heidarzadeh‐Esfahani and colleagues evaluated dietary patterns in relation to total fat intake [76].

#### 4.6.11 Liver

Four SRs examined outcomes related to liver function (*liver enzymes, liver fat, liver echogenicity*), *glucose metabolism* (including *insulin resistance*) and *blood lipids*, in adults and children with non‐alcoholic fatty liver disease (NAFLD, recently renamed to metabolic dysfunction‐associated steatotic liver disease (MASLD)) [72, 103, 133] and healthy adults [85], in relation to total fat intake. The percentage of relevant included studies ranged from 10% to 46.7%. Three SRs evaluated dietary patterns in relation to total fat intake [72, 85, 133], while the other SR compared the highest total fat intake to the lowest [103].

#### 4.6.12 Respiratory

One included SR, published in 2024, examined the association between total fat intake and *type 2 (T2) inflammatory markers* in adolescents and adults with asthma [94]. This SR was partially aligned, with only one study (1.7%) having relevance for the question of this scoping review. For total fat intake analyses, the authors compared the highest category of total fat intake to the lowest category.

#### 4.6.13 Endocrine

A single included SR, published in 2022, evaluated the association between total fat intake and the onset of natural menopause [75]. A total of 36.8% of its included studies were relevant for the scoping review question. For total fat intake analyses, the authors did not specify the analysis of total fat.

#### 4.6.14 Pregnancy

One included SR was published in 2023 and evaluated the association between total fat intake and *oxidative stress markers* in pregnant women [74]. This SR was partially relevant with 30.8% of its included studies relevant to the scoping review question. For total fat intake analyses, the authors compared the highest total fat category to the lowest total fat intake category.

## 5 Discussion

### 5.1 Overview of the Evidence Base

This scoping review aimed to comprehensively explore the breadth and depth of evidence provided by SRs examining the relationship between total fat intake and a broad range of health topics, excluding measures of unhealthy weight gain, or acute postprandial effects. While the relationships between total fat intake and measures of unhealthy weight gain is established, our scoping review highlights the multifaceted relationships between total fat intake and other health outcomes that extend beyond unhealthy weight gain [3].

Health outcomes reported by eight *fully* aligned SRs (i.e., all included primary studies examined total fat intake) were grouped according to broad health topics, mainly including NCDs such as cancer (gastrointestinal, hematological), bone and joint health, gastrointestinal disorders (e.g., inflammatory bowel disease), cardiometabolic outcomes, and cognitive and neurological health (e.g., Alzheimer's disease, dementia).

Only a proportion of primary studies included in 59 *partially* aligned SRs examined the effects of total fat intake; however, the outcomes reported by these studies covered a broader array of health topics related to NCDs, including additional cancer types (e.g., breast, gynecological, urological, liver, skin), as well as respiratory (e.g., asthma), or liver disorders (e.g., NAFLD/MASLD). The predominant NCD focus of SRs investigating, among others, total fat intake and the risk of health outcomes in general populations is also reflected in an earlier scoping review [134]. Only three SRs (*fully* aligned SR *n* = 1; *partially* aligned SR *n* = 2) in our evidence base reported health outcomes not related to NCDs, including outcomes related to endocrine function in men or women, or markers of oxidative stress in pregnancy.

Almost one third of included SRs in our evidence base reported outcomes related to cardiometabolic risk and CVD (n = 2 *fully* aligned; *n* = 19 *partially* aligned), highlighting the potential contribution of total dietary fat intake to the global burden of several leading causes of death over the past three decades [135].

Our review also included four SRs (*n* = 1 *fully* aligned; *n* = 3 *partially* aligned) reporting outcomes related to the risk of dementia and Alzheimer's disease and three *partially* aligned SRs reporting outcomes related to the risk of colorectal and gastric cancer. These NCDs were also leading causes of global deaths between 1990 and 2021 [135].

### 5.2 Overlap of the Evidence Base

Five health topic categories were represented by both *fully* and *partially* aligned SRs (Cancer‐gastrointestinal; Cardiometabolic and Cardiovascular, Cognitive and Neurological, Endocrine, Gastrointestinal). Unique health topics were introduced by both types of SR: one health topic was exclusively represented in one *fully* aligned SR, while nine health topics were exclusively informed by *partially* aligned SRs.

Within the five health topics represented by both *fully* and *partially* aligned SRs, *partially* aligned SRs contributed a considerable number of unique primary studies to the evidence base of health topics addressed by *fully* aligned SRs. We explored whether the limited overlap of the primary evidence base within topics is due to *fully* aligned SRs generally being published earlier than *partially* aligned SRs, and that the former therefore predate the primary studies included in the latter. However, this was not the case: for overlapping health topics, between 80% and 100% of the primary studies included in *partially* aligned SRs were already published at the time of publication of the *fully* aligned SRs in each category. This reflects that, even within similar health topics, *partially* aligned SRs are asking broader review questions, mostly dealing with multiple exposures or interventions, and therefore required more inclusive approaches as compared with *fully* aligned SRs.

### 5.3 Gaps in the Evidence Base

The research gaps described by authors of *fully* aligned SRs were summarized in our review. SR authors identified the need for high‐quality RCTs and studies with prospective designs investigating the relationship between long‐term dietary total fat interventions or exposures and health outcomes other than unhealthy weight gain.

Importantly, SR authors recommended that future primary studies should aim to improve the validity of reported outcomes by adequate adjustment for dietary confounders (e.g., total energy intake, red/processed meat). They also identified inconsistent definitions and variability in how total fat intake was measured, which was reported as a major methodological challenge with recommendation for the use of standardized measurements of fat intake. SR authors also indicated that future primary research should address the lack of geographic and demographic diversity in the current evidence base and include age‐ and sex‐specific analyses for developing dietary guidance across the lifespan.

Although we did not assess the country setting of primary studies included in *partially* aligned SRs, the majority of *partially* aligned SRs did not exclude studies based on their geographical setting. However, for *fully* aligned SRs, a key observation is the concentration of studies conducted in high‐income countries, particularly in North America and Europe. Only one *fully* aligned SR included one study from Africa, despite no methodological exclusions, raising concerns about the generalizability of findings to low‐and middle‐income countries, where dietary patterns and health risks may differ significantly. This geographic bias at the primary study level limits the global applicability of existing evidence and underscores the need for more inclusive and equitable research.

The evidence on the relationship between total fat intake and health outcomes other than unhealthy weight gain in children is sparse. This is not unexpected, however, given the dominant focus on health topics related to NCDs, which are more prevalent in adults due to a combination of physiological changes with advancing age, cumulative risk exposure, and the long‐term consequences of lifestyle.

### 5.4 Limitations of the Scoping Review

One of the main limitations of this review relates to how we categorized our evidence base. To structure our analysis and reporting, we categorized SRs by broad health topic. While we acknowledge that this approach may result in heterogeneity within topics, our categorization facilitated structured reporting of the breadth and depth of the evidence base. Comprehensive details are provided in Supporting Information S1: Tables 3 and 4 for those interested in SRs reporting on specific sub‐categories within broader health topics.

In addition, our findings may not be fully independent of unhealthy weight gain as an outcome. The broad range of health topics covered in this scoping review indicates that both primary researchers and systematic reviewers consider total fat intake to be linked with many health outcomes other than unhealthy weight gain. However, overweight and obesity are risk factors for many NCDs [136]. The fact that the preponderance of evidence in our scoping review resorts in categories related to NCDs may be reflecting unhealthy weight gain as a factor on the causal pathway of total fat intake and NCDs. We also did not assess primary evidence directly but relied on the reporting from the authors of included SRs, which may have shortcomings.

An important limitation of this review is the lack of prospective registration of the review protocol. It is also possible that our search may have missed a small number of eligible systematic reviews that use only alternative descriptors for total fat intake in both their titles and abstracts (e.g., “energy density” was not included among our search terms). Furthermore, we included only SRs in English and may have missed relevant studies published in other languages. However, the SRs included in our scoping review may have included some studies in other languages. Finally, we did not assess the methodological quality of the included SRs since this review aimed to scope the evidence base and not to determine effects of interventions or strength of associations between exposures and outcomes.

## 6 Conclusion

This scoping review maps the available evidence on total fat intake and various health outcomes unrelated to unhealthy weight gain or acute postprandial effects. We found a complex and heterogenous body of evidence, with studies spanning a wide range of health topics predominantly relating to NCDs. The scoping review also identifies methodological and conceptual limitations of the evidence base, as described by SR authors, that constrain our understanding of full impact of total fat intake on health outcomes by identifying several gaps, both in terms of methodology as well as geographic and demographic representation.

## Author Contributions

Celeste Naude was responsible for the review conception and design. Electronic searches were conducted by Anel Schoonees and Sarah Gordon. Data collection and analysis was conducted by Sarah Gordon, Marianne Visser and Amanda Brand. Sarah Gordon drafted the first draft of manuscript, critically reviewed and revised by Marianne Visser and Amanda Brand. All authors reviewed and approved the final manuscript.

## Conflicts of Interest

The authors declare no conflicts of interest.

## Supporting information


Supporting File

## Data Availability

The data that support the findings of this study are available from the corresponding author upon reasonable request.
