# Supplementary material for: Evidence on the Relationships Between Total Fat Intake and Health Outcomes Other Than Unhealthy Weight Gain in Adults and Children: A Scoping Review
Source: Cochrane Evid Synth Methods. 2026 Aug 28;4(5):e70104. doi: 10.1002/cesm.70104 (PMC13533145; doi:10.1002/cesm.70104)
Supplement: Supplementary file 1 [file CESM-4-e70104-s001.docx]

**Supplementary tables**

**Table S1. Search strategies**

**Table S2. Table of excluded studies with reasons for exclusion (n=57)**

**Table S3: Characteristics of included studies for fully aligned reviews (n=8)**

**Table S4. Characteristics of included studies for partially aligned reviews (n=59)**

# Table S1. Search strategies

**PubMed**

Searched on 26 January 2024

Date Filter (1 May 2023-December 2024)

#1 (“Dietary Fats”[MeSH Terms] OR “Diet, High-Fat”[MeSH Terms] OR “Diet, Fat-Restricted”[MeSH Terms])

#2 ((“dietary fat*” OR “fat intake” OR “reduced fat*” OR “reducing fat*” OR “low fat*” OR "high fat*" OR "higher fat*" OR "restricted fat*" OR "restricting fat*")[Title/Abstract])

#3 ((“lower fat*” OR “lowered fat*” OR “modified fat*” OR “modifying fat*” OR “total fat*”)[Title/Abstract])

#4 ((“energy from fat” OR “calories from fat” OR “kilojoules from fat”)[Title/Abstract])

#5 #1 OR #2 OR #3 OR #4

#6 (("systematic review" OR meta-analysis OR metaanalysis OR “meta analysis”)[Title/Abstract])

#7 (("synthesised research" OR "synthesized research" OR "research synthesis" OR “synthesized data” OR “synthesised data” OR “data synthesis”) [Title/Abstract])

#8 ((“pooled data” OR “pooling data” OR “pooled data” OR “pooled results” OR “data pooling” OR “data was pooled” OR “data were pooled”)[Title/Abstract])

#9 ("Meta-Analysis" [Publication Type] OR "Network Meta-Analysis"[Mesh])

#10 ("systematic review"[PT] OR “systematic review”[Topic])

#11 #6 OR #7 OR #8 OR #9 OR #10

#12 #5 AND #11

#13 animals[mh]

#14 humans[mh]

#15 #13 NOT #14

#16 #12 NOT #15

**CDSR**

Searched on 13 July 2020

Search 9 May 2023

Date Filter (2020-2023)

Search 26 January 2024

Date Filter (2023-2024)

Search Name:	Total fat scoping review for WHO_other outcomes

Date Run:	13/07/2020 07:56:03

Comment:	13 July 2020

ID	Search	Hits

#1	MeSH descriptor: [Dietary Fats] explode all trees	7363

#2	MeSH descriptor: [Diet, High-Fat] explode all trees	238

#3	MeSH descriptor: [Diet, Fat-Restricted] explode all trees	952

#4	((“dietary fat*” OR “fat intake” OR “reduced fat*” OR “reducing fat*” OR “low fat*” OR "high fat*" OR "higher fat*" OR "restricted fat*" OR "restricting fat*")):ti,ab,kw	9078

#5	((“lower fat*” OR “lowered fat*” OR “modified fat*” OR “modifying fat*” OR “total fat*”)):ti,ab,kw	1538

#6	((“energy from fat” OR “calories from fat” OR “kilojoules from fat”)):ti,ab,kw	499

#7	#1 OR #2 OR #3 OR #4 OR #5 OR #6 in Cochrane Reviews, Cochrane Protocols	76

**Epistemonikos**

Searched on 13 July 2020

Searched on 9 May 2023

Date filter (2020-2023)

Searched on 29 January 2024

Date filter (2023- 2024)

(title:((title:("dietary fat" OR "fat intake" OR "reduced fat" OR "reducing fat" OR "low fat" OR "high fat" OR "higher fat" OR "restricted fat" OR "lower fat" OR "lowered fat" OR "modified fat" OR "modifying fat" OR "total fat" OR "energy from fat" OR "calories from fat" OR "kilojoules from fat" OR "dietary fats" OR "reducing fats" OR "restricted fats" OR "modifying fats" OR "total fats" OR "energy from fats" OR "calories from fats" OR "kilojoules from fats") OR abstract:("dietary fat" OR "fat intake" OR "reduced fat" OR "reducing fat" OR "low fat" OR "high fat" OR "higher fat" OR "restricted fat" OR "lower fat" OR "lowered fat" OR "modified fat" OR "modifying fat" OR "total fat" OR "energy from fat" OR "calories from fat" OR "kilojoules from fat" OR "dietary fats" OR "reducing fats" OR "restricted fats" OR "modifying fats" OR "total fats" OR "energy from fats" OR "calories from fats" OR "kilojoules from fats"))) OR abstract:((title:("dietary fat" OR "fat intake" OR "reduced fat" OR "reducing fat" OR "low fat" OR "high fat" OR "higher fat" OR "restricted fat" OR "lower fat" OR "lowered fat" OR "modified fat" OR "modifying fat" OR "total fat" OR "energy from fat" OR "calories from fat" OR "kilojoules from fat" OR "dietary fats" OR "reducing fats" OR "restricted fats" OR "modifying fats" OR "total fats" OR "energy from fats" OR "calories from fats" OR "kilojoules from fats") OR abstract:("dietary fat" OR "fat intake" OR "reduced fat" OR "reducing fat" OR "low fat" OR "high fat" OR "higher fat" OR "restricted fat" OR "lower fat" OR "lowered fat" OR "modified fat" OR "modifying fat" OR "total fat" OR "energy from fat" OR "calories from fat" OR "kilojoules from fat" OR "dietary fats" OR "reducing fats" OR "restricted fats" OR "modifying fats" OR "total fats" OR "energy from fats" OR "calories from fats" OR "kilojoules from fats"))))

*Filter: Systematic Review*

**TRIP**

Searched on 13 July 2020

Searched on 23 May 2023

Systematic Review filter

Date filter (2020-2023)

Searched on 29 January 2024

Systematic Review filter

Date filter (2023-2024)

“dietary fat” or “fat intake” or “reduced fat” or “reducing fat” or “low fat” or “high fat” or “higher fat” or “restricted fat” or “lower fat” or “lowered fat” or “modified fat” or “modifying fat” or “total fat” or “energy from fat” or “calories from fat” or “kilojoules from fat” or “dietary fats” or “reducing fats” or “restricted fats” or “modifying fats” or “total fats” or “energy from fats” or “calories from fats” or “kilojoules from fats”

# Table S2. Table of Excluded studies with reasons for exclusion (n=57)

| **Reference** | **Reason for exclusion** |
| --- | --- |
| Abbasnezhad 2020 (11) | Wrong intervention |
| Abboud 2021 (12) | Wrong intervention |
| Ahmad 2023 (13) | Wrong intervention (high fat diet) |
| Alarim 2020 (14) | Wrong intervention (high fat diet) |
| Angelidi 2022 (15) | Wrong intervention (high fat diet) |
| Apekey 2022 (16) | Wrong intervention (high fat diet) |
| Appuhamy 2014 (17) | Wrong intervention (diet and exercise combined) |
| Atabilen 2023 (18) | Wrong intervention (high fat diet) |
| Balk 2015 (19) | Wrong intervention (diet and exercise combined) |
| Bennett 2022 (20) | Wrong intervention |
| Bertrand 2017 (21) | Wrong study design (not a systematic review) |
| Bianchi 2021 (22) | Wrong comparison (no total fat comparison) |
| Boushey 2020 (23) | Wrong comparison (no total fat comparison) |
| Chai 2023 (24) | Wrong intervention (high fat diet) |
| Chawla 2020 (25) | Wrong outcomes (weight loss) |
| Da Costa Brum 2022 (26) | Wrong comparison (no total fat comparison) |
| Dianatinasab 2022 (27) | Wrong comparison (no comparative analysis of total fat) |
| Dietary Guidelines Advisory Committee 2020 (28) | Wrong comparison (no total fat comparison) |
| Filippou et al., 2021 (29) | Wrong intervention (high fat diet) |
| Gabriel 2023 (30) | Wrong outcomes (weight loss) |
| Garcêz 2021 (31) | Wrong comparison (no total fat comparison) |
| Gravesteijn 2022 (32) | Wrong comparison (no total fat comparison) |
| Guerra 2022 (33) | Wrong intervention (high fat diet) |
| Gupta 2022 (34) | Wrong comparison (no total fat comparison) |
| Hooper 2016 (35) | Wrong publication type: summary of original review published before 2014 |
| Hooper 2020 (2) | Wrong outcomes (weight loss) |
| Jaramillo 2023 (36) | Wrong comparison (no total fat comparison) |
| Kaviyarasan 2022 (37) | Wrong intervention (high fat diet) |
| Khodarahmi 2016 (38) | Wrong outcomes (post-prandial) |
| Kiesswetter 2023 (39) | Wrong comparison (no total fat comparison) |
| Lei 2022 (40) | Wrong intervention (high fat diet) |
| Li 2021 (41) | Wrong intervention (high fat diet) |
| Mayer-Davis 2020 (42) | Wrong comparison (no total fat comparison) |
| Muth 2021 (43) | Wrong comparison (no total fat comparison) |
| Nelson 2020 (44) | Wrong comparison (no total fat comparison) |
| Panagiotou 2023 (45) | Wrong comparison (no total fat comparison) |
| Prado 2023 (46) | Wrong intervention (high-fat diet) |
| Rosqvist 2020 (47) | Wrong intervention (high fat diet) |
| Sabo 2023 (48) | Wrong intervention (no total fat) |
| Sabo 2023 (48) | Wrong publication type: duplicate |
| Sanlier 2020 (49) | Wrong study design (not systematic review) |
| Santos 2023 (50) | Wrong comparison (no total fat comparison) |
| Schoeler 2023 (51) | Wrong comparison (no comparative analysis of total fat) |
| Schwab 2021 (52) | Wrong comparison (no comparative analysis of total fat) |
| Sindler 2024 (53) | Wrong intervention (high fat diet) |
| Tarabanis 2023 (54) | Wrong comparison (no total fat comparison) |
| Treadwell 2023 (55) | Wrong comparison (no comparative analysis of total fat) |
| Uhomoibhi 2022 (56) | Wrong intervention (high-fat diet) |
| Urzì 2023 (57) | Wrong study design (not a systematic review) |
| Wang 2022 (58) | Wrong comparison (no total fat comparison) |
| Willems 2021 (59) | Wrong outcomes (weight loss) |
| Xu 2020 (61) | Wrong study design (protocol) |
| Xu 2024 (60) | Wrong intervention (high fat diet) |
| Yang 2017 (62) | Wrong study design (not a systematic review) |
| Yang 2022 (63) | Wrong outcomes (weight loss) |
| Yao 2022 (64) | Wrong comparison (no comparative analysis of total fat) |
| Zhao 2022 (65) | Wrong outcomes (weight loss) |

# Table S3: Characteristics of included studies for fully aligned reviews (n=8)

| **First author and year of publication** | **Aim/objective** | | **Eligible study designs** | **Eligible participants** | **Comparison (treatment /exposure and comparator)** | **Duration of intervention/ follow-up period** | **Pre-specified outcomes** | **No. of studies and study designs included that examined total fat intake**  **Countries** |
| --- | --- | --- | --- | --- | --- | --- | --- | --- |
|  | | **Cancer –gastrointestinal (n = 1)** | | | | | | |
| Shen 2015 (103) | To clarify whether total fat intake is associated with pancreatic cancer risk | | Cohort studies, case-control and nested case-control studies | Inclusion: NS  Exclusion: NS | Highest category of total fat intake compared to lowest category of total fat intake | 8 to 22 years | - Pancreatic cancer | 6 cohorts, 13 case-control studies (1 study conducted in women, 2 conducted in men)  Australia, Canada, China, Finland, Greece, Italy, Japan, Netherlands, Poland, USA |
|  | | **Cancer-haematological (n = 1)** | | | | | | |
| Han 2017 (104) | To quantitatively assess the association between fat consumption and the risk for NHL | | Cohort studies, case-control and nested case-control studies | Inclusion:NS  Exclusion: NS | Highest category of total fat intake compared to lowest category of total fat intake | NR | - NHL | 2 cohort studies, 8 case-control studies (1 study conducted in males, 4 conducted in females)  Canada, Italy, Oman, Sweden, USA |
|  | | **Bone and joint (n = 1)** | | | | | | |
| Mozaffari 2018 (105) | To quantify the association between dietary intakes of total fat, SFAs, and MUFAs with the risk of fracture | | Observational studies | Inclusion: Adults (18 years or older)  Exclusion: NS | Highest category of total fat intake compared to lowest category of total fat intake | mean (range): 7.6 to 8.9 years | - Fracture risk | 4 prospective cohort studies; 3 case-control studies (3 studies conducted in women)  China, Europe, Spain, Sweden, USA |
|  | | **Cardiometabolic and cardiovascular (n = 2)** | | | | | | |
| Lu 2018 (106) | To summarise the impact of low-fat compared to a high-fat diets on cardiometabolic risk factors in metabolically healthy people | | RCTs | Inclusion: Metabolically healthy adults  with BMI ≥ 25 kg/m2; Exclusion: Those using drugs with potential influence on any review outcome | Low-fat diet (<30% of total energy), compared to high-fat diet (≥30% of total energy); Excluded: high-fat diet with 30% of the total energy only. Diets that replaced fat with artificial fat. | 6 weeks to 2 years | - SBP - DBP - LDL-C - HDL-C - TC - TAG - insulin - glucose - leptin - total adiponectin - Hs-CRP | 20 RCTs (5 RCTs conducted in women)  Australia, Canada, Denmark, Germany, Spain, Sweden, USA |
| Schwingshackl 2014 (107) | To examine the long-term effects of high-fat (HF) v. low-fat (LF) diet consumption on the indicators of glycaemic control as well as cardiovascular risk factors in pre-diabetic and diabetic individuals | | RCTs | Inclusion:  Adults (aged 18 years and older) with either impaired FG levels (>5·6 mmol/l) or insulin resistance or type 2 diabetes  Exclusion: NS | High fat intake (> 30% of total energy), compared to Low fat intake (≤ 30% of total energy) | 12 months to 6 years | - TC - LDL-C - HDL-C - TAG - SBP - DBP - CRP - FI - FG - Glycosylated haemoglobin - Adiponectin | 14 RCTs  NR |
|  | | **Cognitive and neurological (n = 1)** | | | | | | |
| Ruan 2018 (108) | To assess the association between dietary fat intake and risk of AD and dementia | | Prospective cohort studies | Inclusion: Healthy adults  Exclusion: NS | Highest category of total fat intake compared to lowest category of fat intake | 2.1 to 21 years | - AD - Dementia | 4 prospective cohort studies  Finland, Netherlands, USA |
|  | | **Gastrointestinal (n = 1)** | | | | | | |
| Wu 2016 (109) | To assess the association between dietary fat intake and IBD (UC and CD) risk | | Cohort, case-control or cross-sectional studies | Inclusion: NS  Exclusion: NS | Highest category of total fat intake compared to lowest category of total fat intake | NR | - IBD (UC, CD) | 3 cohort studies, 5 case-control studies  Canada, Europe, France, Israel, Japan, Netherlands, Sweden |
|  | | **Endocrine (n = 1)** | | | | | | |
| Whittaker 2021 (110) | To investigate the association between Low Fat diets and testosterone and related hormone markers related hormonal markers. | | NS | Inclusion:  Healthy adult male participants only (18+ years)  Exclusion: NS | A difference in fat intake between diets ≥10% of total energy to provide a meaningful distinction between diets on the basis of fat intake. Percentage rather than total fat intake was used to account for participant differences in energy intake requirements. | 2 to 10 weeks | - Total testosterone - Free testosterone - Urinary testosterone - Dihydrotestosterone - Sex hormone binding globulin (SHBG) - Luteinising hormone | 6 intervention studies  Finland, South Africa, USA |
| Abbreviations: AD, Alzheimer’s disease; CD, Crohn’s disease; DBP, diastolic blood pressure; FG, fasting glucose; FI, fasting insulin; HDL-C, high-density lipoprotein cholesterol; Hs-CRP, high-sensitivity C-reactive protein; IBD, inflammatory bowel disease; LDL-C, low-density lipoprotein cholesterol; NHL, non-Hodgkin lymphoma; SBP, systolic blood pressure; TAG, triacylglycerol; TC, total cholesterol; UC, ulcerative colitis | | | | | | | | |

# Table S4. Characteristics of included studies for partially aligned reviews (n=59)

| **First author and year of publication** | **Aim/objective** | **Eligible study designs** | **Eligible participants** | **Duration of intervention / follow-up period** | **Pre-specified outcomes reported** | **Total number of included studies** | **No. of studies (%) and study designs included that examined total fat intake** |
| --- | --- | --- | --- | --- | --- | --- | --- |
| **Cancer - breast (n = 6)** | | | | | | | |
| Becerra-Tomás 2023 (66) | This work aimed to systematically review and meta-analyse the accumulated evidence on postdiagnosis diet (foods, food groups, dietary patterns, food components, nutrients, and dietary supplements) and breast cancer outcomes | RCTs, longitudinal observational studies or pooled analyses | Inclusion: NS  Exclusion: NS | 5 to 7.12 years | - Mortality (All-cause; Breast cancer specific; CVD) - Breast cancer recurrence - Other primary cancer risk | 108 | 3 (2.7%) RCTs |
| Bu 2023 (112) | To systematically review the effects of dietary patterns on breast cancer risk, prognosis, and quality of life in survivors. | Cohort study, RCT, cross-sectional research, or case control | Inclusion: NS  Exclusion: any population not explicitly defined as cancer survivors; cell and animal  experiments | NR | - Breast cancer risk - Mortality (All cause; cause specific) - Breast cancer recurrence - Quality of life | 59 | 2 (3.4%) RCTs |
| Cao 2016 (86) | To assess the association between dietary intakes of total fat and fatty acids and breast cancer risk | Prospective studies and nested case-control studies | Inclusion: NS  Exclusion: NS | 2 to 25 years | - Breast cancer risk | 24 | 20 (83.3%): 15 prospective cohort studies, 5 nested case-control studies |
| Gopinath 2022 (111) | This review aims to study the effect dietary fat has on the risk of breast cancer incidence as well as its influence on the survival of breast cancer patients. The main population under consideration for this review is women older than the age of 18. | NS | Inclusion: Women over the age of 18, Exclusion: Studies involving males and animals | 6 months to 8.5 years | - Breast cancer risk - Survival | 22 | 2 (9.1%) RCTs |
| Mourouti 2015 (78) | To evaluate the evidence about the relationship between specific foodstuffs and breast cancer risk | RCTs, cohort and, case-control studies | Inclusion: NS  Exclusion: Women during adolescence | 3 to 20 years | - Breast cancer risk | 140 | 21 (15%): 2 RCTs, 9 cohort and 10 case-control studies |
| Wu 2015 (83) | To investigate the correlation between dietary factors and breast cancer in Chinese women | RCT, NRCT, cohort, case-control studies | Inclusion: Chinese women  Exclusion: NS | NR | - Breast cancer risk | 22 | 9 (41%) case-control studies |
| **Cancer – gastrointestinal (n = 4)** | | | | | | | |
| Han 2015 (113) | To summarise the evidence regarding the association between dietary fat intake and gastric cancer risk | Case-control and prospective cohort studies | Inclusion: NS  Exclusion: NS | NR | - Gastric cancer risk | 22 | 21 (95%): 1 prospective cohort study, 20 case-control studies |
| He 2017 (114) | To evaluate the role of fat intake in the development of esophageal adenocarcinoma (EAC) and esophageal squamous cell carcinoma (ESCC) | Case-control and prospective cohort studies | Inclusion: NS  Exclusion: NS | NR | - EAC risk - ESCC risk | 19 | 12 (63.2%): 1 prospective cohort study, 11 case-control studies |
| Kim 2018 (115) | To evaluate the role of fat intake in the development of colorectal cancer | Case-control and prospective cohort studies | Inclusion: NS  Exclusion: NS | 3.3 to 32 years | - Colorectal cancer risk | 18 | 11 (61.1%) prospective cohort studies |
| Vahid 2021 (116) | To identify the major nutritional risk factors for gastric cancer | NS | Inclusion: Patients with gastric cancer  Exclusion: no cell or animal studies | NS | - Gastric cancer risk - Morbidity - Death - Complications | 85 | 1 (1.2%) cohort |
| **Cancer – gynaecological (n = 6)** | | | | | | | |
| Crane 2014 (68) | To evaluate the role of diet in ovarian cancer risk | Prospective cohort studies (with ≥ 200 ovarian cancer cases) | Inclusion: Adult females  Exclusion: NS | 8.1 to 9 years | - Ovarian cancer risk | 24 | 2 (8.3%) prospective cohort studies |
| Hou 2015 (117) | To assess the evidence of associations between dietary fat epithelial ovarian cancer risk (EOC) | Observational studies | Inclusion: NS  Exclusion: NS | NS | - EOC risk | 20 | 14 (70%): 4 cohort studies and 10 case-control studies |
| Qiu 2016 (118) | To analyse the association of dietary fat consumption with risk of ovarian cancer | Cohort and case-control studies | Inclusion: Women 18 years or older  Exclusion: NS | NR | - Ovarian cancer risk (including EOC subtypes) | 25 | 17 (68%): 6 cohort studies and 11 case-control studies |
| Sadeghi 2019 (91) | To assess the association between the consumption of the different type of fatty acids and the risk of ovarian cancer | Cohort studies, case-control and nested case-control studies, cross-sectional studies | Inclusion: Women (18 years or older)  Exclusion: NS | 9 to 28 years | - Ovarian cancer risk | 21 | 13 (62%): 5 cohort studies and 8 case-control studies |
| Zhao 2016 (120) | To evaluate the associations between dietary fat intake and endometrial cancer risk | RCT, cohort and case-control studies | Inclusion: NS  Exclusion: NS | 1 month to 26 years | - Endometrial cancer | 21 | 16 (76%): 5 cohort studies and 11 case-control studies |
| Zhang 2023 (119) | To assess the key dietary fats and serum lipids that increased the risk of ovarian cancer. | Observational studies with cohort, case–control or nested case– control | Inclusion: NS  Exclusion: NS | NR | - Ovarian cancer risk | 41 | 17 (41.5%): 7 cohort studies, 10 case control studies |
| **Cancer – liver (n = 1)** | | | | | | | |
| Zhao 2021 (97) | To quantify the associations between dietary fats and liver cancer risk | Inclusion: the study design was cohort design or nested case-control design, or case-cohort design; | Inclusion: NS  Exclusion: NS | 11.4 years to 26.6 years | - Liver cancer risk (primary liver cancer, hepatocellular carcinoma or   intrahepatic bile duct carcinoma) | 14 | 5 (35.7%) cohort studies |
| **Cancer – skin (n = 1)** | | | | | | | |
| Ruan 2020 (90) | To evaluate the relationship between different types of fats consumption and the risk of three major types of skin cancer | Cohort and case-control studies | Inclusion: NS  Exclusion: NS | 10 to 28 years (cohort studies) | - Skin cancer risk (basal cell carcinoma (BCC), squamous cell carcinoma, (SCC) and cutaneous malignant melanoma (CMM) | 12 | 10 (83.3%): 2 cohort studies, 8 case-control studies |
| **Cancer – urological (n = 2)** | | | | | | | |
| Wang 2019 (96) | To understand the association between dietary fat intake and bladder cancer risk | Observational studies | Inclusion: NS  Exclusion: NS | NR | - Bladder cancer risk | 10 | 9 (90%): 2 cohort studies, 7 case-control studies |
| Xu 2015 (121) | To investigate the relationship between the consumption of different types of fats and the risk for prostate cancer | Cohort and case-control studies | Inclusion: NS  Exclusion: NS | 2 to 17.4 years | - Prostate cancer risk | 14 | 13 (92%) prospective cohort studies |
| **Cancer – overall (n = 1)** | | | | | | | |
| Rinnienella 2020 (122) | This review aimed to investigate the associations between post-diagnostic diet and prognostic outcomes in cancer patients | Prospective or retrospective cohort studies. | Inclusion: adult (>18 years old) patients diagnosed with breast, gastrointestinal (gastric,  pancreatic, colorectal), gynaecological (uterine, cervical, ovarian, endometrial, vulvar), lung and  urological (prostate, bladder) cancers. Exclusion: patients with other types of cancer | 18 years | - Survival (Overall, Disease free, Recurrence free) - Mortality (All-cause; Cancer-specific; cause other than specific cancer) - Cancer progression/   recurrence | 29 | 1 (3.4%) cohort study |
| **Respiratory (n = 1)** | | | | | | | |
| Visser 2024 (93) | To review the effect of dietary interventions on markers of T2 inflammation in adults with asthma. | RCTs, quasi-experimental studies, before-after studies,  cohort studies, case-control studies and cross-sectional studies | Inclusion: adults and adolescents (≥12 years of age) with asthma were included.  Exclusion:  pregnancy, infants or children (<12 years of age) | 6 months | T2 inflammatory markers | 59 | 1 (1.7%): 1 RCT |
| **Cardiometabolic and cardiovascular (n = 19)** | | | | | | | |
| Barkas 2020 (123) | To investigate the impact of cholesterol-lowering diet and other dietary interventions on the incidence or mortality of cardiovascular disease and lipid profile of patients with familial hypercholesterolemia | RCTs and trials using quasi-randomization methods were  alternatively included in case of sufficient evidence showing that treatment and comparison groups  were comparable in terms of clinical and nutritional status | Inclusion: Children and adults with FH (alternative named as inherited dyslipidaemia)  Exclusion: NS | 8 weeks | - Mortality (CVD-specific) - CVD - CHD - Stroke - Peripheral arterial disease - TC - TG - HDL-C - LDL-C - VLDL-C - Apo A-1 - Apo B - Lp(a) | 17 | 1 (5.9%) RCT |
| Brayner 2023 (67) | This scoping review aimed to systematically search and synthesize the literature regarding the association between dietary patterns characterized by dietary fats and T2D risk using reduced rank regression | Observational studies (cross-sectional and cohort studies) | Inclusion: Human  Exclusion: NS | NS | - T2DM risk - Insulin (including resistance) - Glucose - HbA1c - Gestational diabetes | 8 | 3 (37.5%) 2 prospective, 1 cross-sectional study |
| Dietary Guidelines Advisory Committee 2020 (70) | Assess the relationship between dietary patterns consumed and risk of cardiovascular disease | RCT, non-RCT, including quasi-experimental and controlled before and after studies, prospective cohort studies, retrospective cohort studies, nested case-control studies. | Inclusion: Human participants (Males, Females, Women during pregnancy and lactation.  Exclusion: Non-human participants (e.g., animal or in-vitro models). | NS | - TC - TG - LDL-C - HDL-C - BP - MI - CHD - Coronary artery disease - Congestive heart failure - Peripheral arterial disease - Stroke - Venous thrombosis - Mortality (CVD-specific) | 350 | 14: 9 (1.4%) RCTs, 5 cohort studies |
| Jayedi 2023 (100) | Systematic review coupled with pairwise and dose-response meta-analyses of dietary intake and biomarkers of fats and fatty acids interventions and risk of coronary events in adults. | Prospective observational design including prospective cohort studies, case-cohort, and nested case-control studies; | Inclusion: Adults aged ≥18 years regardless of their cardiovascular disease condition  Exclusion: NS | 5 years to 30 years | - Mortality (CVD-specific) - Acute coronary syndrome - MI - CHD - Ischaemic heart disease - Angina | 165 | 13 (7.9%) observational studies |
| Jing 2023 (126) | The aim of this network meta-analysis was to compare the impacts of various dietary approaches on the glycaemic control of T2DM patients | RCTs | Inclusion: age >18 years; T2DM Exclusion: pregnant women, children, and adolescents, patients with abnormal glucose metabolism, chronic kidney disease, and disordered eating patterns | 6 months to 36 months | - HbA1c - Glucose | 42 | 7 (16.6%) RCTs |
| Julibert 2019 (87) | To assess evidence for the effect of dietary fat intake on MetS occurrence and reversion in adults | RCTs, observational studies | Inclusion: Adults  Exclusion: NS | 3 months to 6 years | - MetS | 30 | 9 (30%) RCTs |
| Karam 2023 (101) | To determine the relative efficacy of structured named  diet and health behaviour programmes (dietary  programmes) for prevention of mortality and major  cardiovascular events in patients at increased risk of  cardiovascular disease | RCT | Inclusion: Adults at risk of cardiovascular disease with two or more established risk factors for cardiovascular disease (e.g., hypertension, dyslipidaemia, obesity, diabetes mellitus), or established cardiovascular disease (history of coronary artery disease, myocardial infarction, stroke,  or peripheral artery disease).  Exclusion: NS | 0.75 years and 17 years | - Mortality (all-cause, CVD-specific) - Stroke - MI - Angina - Heart failure - Peripheral arterial disease - Atrial fibrillation - Unplanned CV interventions | 40 | 24 (60%) RCTs |
| Keshani 2024 (125) | This systematic review with meta-analysis was undertaken to evaluate the effects of TDs diet on blood lipids, apolipoprotein A-1, apolipoprotein B, blood pressure, fasting blood glucose, and insulin | RCT, Non-RCT, crossover trial, parallel trial and clinical triail | Inclusion: Any population aged >18 years  Exclusion: NS | 28 days to 30 weeks | - TC - TG - LDL-C - HDL-C - ApoB - ApoA-1 - BP - Glucose - Insulin | 34 | 16 (47.1%) 10 quasi-randomized trials, 6 RCTs |
| Kim 2020 (124) | Previous studies have separately reported the contributions of dietary factors to the risk of cardiovascular disease (CVD) and its markers, including blood pressure (BP) and lipid profile. This study systematically reviewed the current evidence on this issue in the Korean population. | NS | Inclusion: Korean population  Exclusion: NS | NS | - CVD - BP - Hypertension - TC - TG - HDL-C - LDL-C | 62 | 3 (4.8%) case control/cross-sectional studies |
| Kim 2021 (88) | The association between dietary fat and mortality remains inconsistent, and recent results for the association between dietary saturated fat and chronic disease are controversial. To quantitatively assess this association, we conducted a meta-analysis of prospective cohort studies. | Observational studies, prospective cohort studies (prospective in design) | Inclusion: NS  Exclusion: Participants with preexisting disease at baseline | 6.6 years to 32 years | - Mortality (all-cause, CVD- and cancer-specific) | 19 | 11 (57.9%) prospective cohort studies |
| Leed 2023 (74) | The purpose of this review was to determine whether various dietary factors were associated with arterial stiffness in the pediatric population | Cross-sectional, cohort or intervention study design | Inclusion: Children or adolescents (< 18 years).  Exclusion:  participants with a chronic or genetic disease (e.g., type 1 diabetes) | 19 | - Arterial stiffness | 19 | 2 (10.5%) prospective cohort studies |
| Liang 2022 (75) | The network meta-analysis (NMA) was conducted to compare and rank the effects of different dietary patterns on NCD biomarkers, and associations of dietary patterns’ underlying macronutrient composition with NCD biomarkers were determined by a nutritional geometry approach | RCTs | Inclusion: Healthy adult participants Exclusion: (1) all participants had a current diagnosis of metabolic syndrome, diabetes, cardiovascular diseases, cancers, or other diseases; (2) study participants were pregnant or breastfeeding; | NR | - NCD risk biomarkers | 59 | 4 (6.7%) RCTs |
| Luong 2022 (76) | To consolidate the current evidence on the short- and long-term effects of dietary patterns on cardiometabolic health in adults aged >65 years. | RCTs, cluster RCTs, pseudorandomized controlled trial, nonrandomized controlled clinical trials, cluster trials, prospective cohort studies, retrospective cohort studies, interrupted time-series studies with a control group, case-control studies, and nested case-control studies. | Inclusion: Adults >65 y who are healthy, at risk of cardiometabolic disease, diagnosed with cardiometabolic disease, and those with other underlying disease(s).  Exclusion: Adults with any acute or chronic disease that inherently affects dietary intake such as those with T1D, bariatric surgery, kidney disease and receiving dialysis, cancer, food allergies, food intolerances, and eating disorders. | 8 years | - Mortality (CVD- or metabolic disease-specific) - CVD - CHD - Stroke - MI - T2DM - Hypertension - MetS - BP - Glucose - HbA1c - Insulin (including resistance/sensitivity/secretion) - TC - TG - HDL-C - LDL-C | 16 | 1 (6.3%) RCT |
| Ma 2024 (77) | Systematic review and dose-response meta-analysis of prospective cohort studies to summarize the association of dietary macronutrient intake with all-cause and cause-specific mortality and CVD morbidity. | Inclusion: prospective cohort study. | Inclusion: Adults  Exclusion: NS | 2.2 years to 30 years | - Mortality (all-cause, CVD- and cancer-specific) - CVD events | 101 | 53 (52.5%) prospective cohort studies |
| Mazidi 2020 (89) | To examine the association between fat consumption (total, MUFA, PUFA, SFA) and all-cause and cause-specific (CHD, stroke and T2DM) mortality | Prospective cohort studies | Inclusion: Adults  Exclusion: NS | 6 to 32 years | - Mortality (all-cause, CHD-, stroke- and T2DM-specific) | 29 | 10 (34%) prospective cohort studies |
| Schwab 2014 (92) | To assess the effects of total fat intake and various combinations and proportions of fatty acid classes in the diet, on indicators of clinical outcomes | RCTs, observational studies (prospective cohort studies, nested or retrospective case-control studies) | Inclusion: Healthy adults (aged 18 to 70 years), those with dyslipidemia, glucose intolerance, or overweight (BMI >30) Exclusion: NS | 6 months to 22 years (CVD risk outcomes)  NR (cancer outcomes) | - Blood lipids - Glucose - Insulin - BP - Inflammatory markers - T2DM - CVD - Cancer - All-cause mortality | 607 | 57 (9.4%): 8 RCTs, 33 cohort studies, 16 case-control studies) |
| Swedish Agency for Health Technology Assessment and Assessment of Social Services 2022 (127) | The aim of this project was to evaluate the positive and negative health effects of food and dietary treatments in individuals with type 1, type 2, and gestational diabetes, and additionally to analyse health economic and ethical aspects. The report will serve as a scientific basis for the development of new recommendations for dietary treatment in diabetes by the Swedish National Board of Health and Welfare | RCTs  and prospective cohort studies | Inclusion: Adults, from 18 years with diabetes, type 1 and 2. Women from 18 years with gestational diabetes.  Exclusion: NS | 12 months | - Mortality (all-cause and cause-specific) - Total morbidity - Diabetes complications - Need for drug treatment - Osteoporosis - T2DM remission - QoL - Hypo- and hyperglycaemia - Pre-eclampsia - Negative pregnancy outcomes - Glucose - HbA1c - BP - TC - TG - LDL-C - HDL-C | 81 | 1 (1.2%) RCT, 2 (2.5%) prospective cohort studies |
| Von Frankenberg 2014 (94) | To investigate  the effects of dietary lipids on circulating adiponectin concentrations in adults | RCTs | Inclusion: Adults  Exclusion: NS | 5 days to 2.8 years | - Adiponectin | 53 | 15 (28%) RCTs (12 in quantitative synthesis and 3 in qualitative synthesis only) |
| Zhu 2019 (99) | To summarise the evidence of the relation of dietary fat intake and CVDs risk, and evaluate the dose-response relationship of CVDs and dietary fat intake | Cohort and nested case-control studies | Inclusion: NS  Exclusion: NS | < 10 years (for 15 studies)  ≥10 years (for 30 studies) | - CVD | 43 | 30 (70%) prospective cohort studies |
| **Pregnancy (n = 1)** | | | | | | | |
| El Sherbiny 2023 (71) | Therefore, the aim of this work is to sum up the existing knowledge to fill the literature gap herein identified, and to verify the extent of the impact that diet can have on OS during pregnancy. Diet is a fundamental actor in the development of the foetus, and nutritional education should be introduced in the routine talk during pregnancy interviews | Observational studies | Inclusion: Pregnant women at any stage of gestation, (≥16 years). Exclusion: studies involving pathological conditions (e.g., gestational diabetes, pre-eclampsia, and hypertension,) or multiple pregnancies | NS | - Oxidative stress biomarkers | 13 | 4 (30.8%): 3 cohort studies, 1 case-control study |
| **Cognitive and neurological (n = 8)** | | | | | | | |
| Attuquayefio 2015 (128) | To review the experimental evidence of the link between diet and cognition in humans | Intervention studies | Inclusion: Adults (18 years and older)  Exclusion: NS | 2 days to 6.5 years | - Changes in cognitive function | 30 | 15 (50%) RCTs |
| Cao 2019 (129) | To examine the associations between different types of dietary fat intake and cognitive outcomes among older populations | Prospective cohort studies | Inclusion: Adults aged ≥ 55 years from general population  Exclusion: NS | 2.1 to 21 years | - Mild Cognitive Impairment - Dementia - Alzheimer’s disease | 9 | 7 (77%) prospective cohort studies |
| Frediani 2024 (131) | This systematic review aims to study the efficacy of dietary interventions, vitamins, and nutritional supplementation in the management of chronic  neuropathic pain in adults. | RCT, prospective cohort studies, or cross-sectional studies | Inclusion: Adults  Exclusion: NS | 20 weeks | - Pain | 40 | 1 (2.5%) RCT |
| Qu 2019 (130) | To summarise the evidence regarding links between fat consumption and PD | Case-control, nested case-control, cohort, and prospective studies | Inclusion: NS  Exclusion: NS | 2 to 14 years | - Parkinson’s Disease | 9 | 6 (67%): 2 cohort studies, 4 case-control studies |
| Snetselaar 2023 (79) | The objective of this study was to assess the efficacy of different dietary approaches on MS-related fatigue and quality of life (QoL) through a systematic review of the literature and network meta analysis (NMA). | RCTs | Inclusion: Adults with definitive MS based on McDonald criteria; Exclusion: trials that included pregnant women or individuals age younger than 18 years | 12 months | - Modified fatigue impact scale - QoL | 11 | 1 (9.1%) RCTs |
| Townsend 2023 (80) | This narrative systematic review aimed to synthesise prospective and intervention study data to evaluate the impact of aposteriori and a-priori derived DPs on cognitive ageing, from cognitive decline to incident dementia | RCTs, prospective studies | Inclusion: NS  Exclusion: NS | 18.7 years | - Cognitive impairment or probable dementia. - Global cognitive function via 3MSE Suspected cognitive impairment assessed further using CERAD and assessment via clinical experts. | 93 | 1 (1.1%) RCT |
| Wang 2015 (81) | To summarise the evidence on macronutrients intake and PD risk | NS | Inclusion: NS  Exclusion: NS | NR | - Parkinson’s Disease | 41 | 12 (29%): 6 prospective cohort studies, 6 case-control studies |
| Zhu 2021 (98) | Systematically search for prospective cohort studies to provide new evidence on relationships between dietary fatty acid intake and cognition and to obtain more reliable conclusions. | Prospective cohort studies | Inclusion: > 40 y of age; normal cognitive state at the beginning of the study,  Exclusion: Patients had a history of heart disease, stroke, diabetes | 2.1 years to 20 years | - Alzheimer’s disease Dementia - Mild cognitive Impairment | 14 | 7 (50%) cohort studies |
| **Endocrine (n = 1)** | | | | | | | |
| Grisotto 2022 (72) | Systematically reviewed and evaluated published research about associations between diet and onset of natural menopause (ONM). | RCTs, Case-cohort studies, prospective cohort studies | Inclusion: Pre and/or peri-menopausal women.  Exclusion: post-menopausal women or women with medical conditions at baseline (e.g., breast cancer, HIV-infected women); reported solely unnatural menopause; and evaluated biomarkers of dietary intake. | 3 to 10 years | - Onset of natural menopause | 19 | 7 (36.8%) prospective cohort studies |
| **Gastrointestinal (n = 4)** | | | | | | | |
| Heidarzadeh-Esfahani 2021 (73) | Systematic review of articles investigating the association between food and dietary patterns with GERD. | Observational studies, including cross-sectional, case-control, prospective, and retrospective studies. | Inclusion: Adults  Exclusion: NS | NS | - Gastro- oesophageal reflux disease risk | 25 | 1 (4%) case control |
| Wang 2017 (95) | To assess the role of fat intake in the development of UC | Cohort studies, case-control and nested case-control studies | Inclusion: NS  Exclusion: NS | 6 months to 5 years | - Ulcerative Colitis risk | 9 | 5 (55%): 2 cohort studies, 3 case-control studies |
| Zeng 2017 (84) | To assess the role of macronutrient intake in the development of CD | Cohort and case-control studies | Inclusion: NS  Exclusion: NS | 5 to 22 years | - Crohn’s disease risk | 9 | 6 (67%): 3 cohort studies, 3 case-control studies |
| Zhang 2021 (85) | Therefore, we reviewed and analysed the current literature to elucidate which dietary and lifestyle factors are associated with GERD, provide specific and informative dietary and lifestyle recommendations for patients, and provide information for follow-up studies. We also attempt to elucidate the dietary and lifestyle differences associated with GERD between Western countries and Eastern countries | Cross-sectional, cohort, case control | Inclusion: Participants ≥18 years with gastro-oesophageal reflux disease (including nonerosive gastroesophageal reflux dis  ease, reflux esophagitis or Barrett’s  esophagus  Exclusion: NS | NS | - Gastro-oesophageal reflux disease risk | 72 | 1 (1.4%) case-control |
| **Liver (n = 4)** | | | | | | | |
| Del Bo 2023 (69) | This systematic review and meta-analysis aimed to evaluate the overall effects of MD intervention in a cohort of NAFLD patients targeting specific markers such as central obesity, lipid profile, liver enzymes and  fibrosis, and intrahepatic fat (IHF). | RCTs | Inclusion: Adult participants (age > 18 years) and overweight and obese adults (BMI >25 kg/m2)  that were diagnosed with NAFLD  Exclusion: NS | 3 months | - Glycemic indices - Liver function - Liver fat content - Fatty liver indices | 10 | 4 (40%) RCTs |
| Katsagoni 2020 (102) | The present systematic review assessed the efficacy of lifestyle (diet or exercise) based on randomized controlled clinical trials (RCTs) on liver, anthropometric, glucose, and lipid parameters in children, with imaging or biopsy-proven NAFL | RCTs | Inclusion: Children with NAFLD, aged below or equal to 18 years old.  Exclusion: other causes for secondary liver fat accumulation | 12 weeks | - Liver enzymes - Liver fat content - Liver echogenicity - Glucose - Insulin (including resistance) - TC - TG, - HDL-C   LDL-C/ox-LDL-C | 10 | 1 (10%) RCT |
| Varkeneh 2022 (132) | The aim of this review is to summarize the results of current randomized controlled trials evaluating the effects of low fat diet (LFD) vs. low carbohydrate diet (LCD) on NAFLD and liver enzymes. | NS | Inclusion: Adult subjects (>18 years of age) with NAFLD. Exclusion: children, pregnant women, other diseases or animals | 4 weeks to 24 weeks | - Liver enzymes | 15 | 7 (46.7%) RCTs |
| Winters-Van Eekelen 2021 (82) | We aimed to assess the effects of dietary macronutrient  composition on liver fat content by conducting a systematic review and meta-analysis of randomized controlled trials in adults. | RCTs | Inclusion: Healthy humans, (pre)diabetes, obesity, metabolic syndrome, CVD and/or NAFL or NASH, Adults (18+ years).  Exclusion: Malignant diseases  Individuals with alcoholic, drug-induced, viral or genetic causes of liver injury | 2 weeks to 26 weeks | Liver fat content | 26 | 6 (23.1%) RCTs |
| **Abbreviations:** BMI: Body Mass Index; BP: blood pressure; CHD: coronary heart disease; CVD: cardiovascular disease; FH: Familial hypercholesterolaemia; HbA1c: glycosylated haemoglobin; HDL-C: High-density lipoprotein cholesterol; HIV: Human Immunodeficiency Virus; HT: Hypertension; LDL-C: Low density lipoprotein cholesterol; Lp (a): Lipoprotein (a); MetS: Metabolic Syndrome; MI: Myocardial infarction; NAFLD: Non-alcoholic fatty liver disease, NASH: Non-alcoholic steatohepatitis; NCD: Non-communicable disease; NS: Not stated, NR: Not reported QoL: Quality of life;; TAG: triacylglycerol; TC: Total cholesterol; T1DM: Type 1 Diabetes Mellitus; T2DM: Type 2 Diabetes Mellitus. | | | | | | | |

# References

2.	Hooper L, Abdelhamid AS, Jimoh OF, Bunn D, Skeaff CM. Effects of total fat intake on body fatness in adults. Cochrane Database of Systematic Reviews. 2020;6(6):Cd013636. doi: 10.1002/14651858.cd013636.

11.	Abbasnezhad A, Falahi E, Gonzalez MJ, Kavehi P, Fouladvand F, Choghakhori R. Effect of Different Dietary Approaches in Comparison with High/Low-Carbohydrate Diets on Systolic and Diastolic Blood Pressure in Type 2 Diabetic Patients: A Systematic Review and Meta-Analysis. Preventive Nutrition and Food Science. 2020;25(3):233-45. doi: 10.3746/pnf.2020.25.3.233.

12.	Abboud M, AlAnouti F, Georgaki E, Papandreou D. Effect of Ketogenic Diet on Quality of Life in Adults with Chronic Disease: A Systematic Review of Randomized Controlled Trials. Nutrients. 2021;13(12):4463. doi: 10.3390/nu13124463.

13.	Ahmad S, Ahmed SB, Khan A, Wasim M, Tabassum S, Haider S, et al. Natural remedies for Alzheimer's disease: A systematic review of randomized controlled trials. Metabolic Brain Disease. 2023;38(1):17-44. doi: 10.1007/s11011-022-01063-9.

14.	Alarim RA, Alasmre FA, Alotaibi HA, Alshehri MA, Hussain SA. Effects of the Ketogenic Diet on Glycemic Control in Diabetic Patients: Meta-Analysis of Clinical Trials. Cureus. 2020;12(10):e10796. doi: 10.7759/cureus.10796.

15.	Angelidi AM, Papadaki A, Nolen-Doerr E, Boutari C, Mantzoros CS. The effect of dietary patterns on non-alcoholic fatty liver disease diagnosed by biopsy or magnetic resonance in adults: a systematic review of randomised controlled trials. Metabolism. 2022;129:155136. doi: 10.1016/j.metabol.2022.155136.

16.	Apekey TA, Maynard MJ, Kittana M, Kunutsor SK. Comparison of the Effectiveness of Low Carbohydrate Versus Low Fat Diets, in Type 2 Diabetes: Systematic Review and Meta-Analysis of Randomized Controlled Trials. Nutrients. 2022;14(20):4391. doi: 10.3390/nu14204391.

17.	Appuhamy JA, Kebreab E, Simon M, Yada R, Milligan LP, France J. Effects of diet and exercise interventions on diabetes risk factors in adults without diabetes: meta-analyses of controlled trials. Diabetology & Metabolic Syndrome. 2014;6:127. doi: 10.1186/1758-5996-6-127.

18.	Atabilen B, Akdevelioğlu Y. Effects of different dietary interventions in multiple sclerosis: a systematic review of evidence from 2018 to 2022. Nutritional Neuroscience. 2023;26(12):1279-91. doi: 10.1080/1028415X.2022.2146843.

19.	Balk EM, Earley A, Raman G, Avendano EA, Pittas AG, Remington PL. Combined Diet and Physical Activity Promotion Programs to Prevent Type 2 Diabetes Among Persons at Increased Risk: A Systematic Review for the Community Preventive Services Task Force. Annals of Internal Medicine. 2015;163(6):437-51. doi: 10.7326/M15-0452.

20.	Bennett G, Bardon LA, Gibney ER. A Comparison of Dietary Patterns and Factors Influencing Food Choice among Ethnic Groups Living in One Locality: A Systematic Review. Nutrients. 2022;14(5):941. doi: 10.3390/nu14050941.

21.	Bertrand KA, Giovannucci E, Rosner BA, Zhang SM, Laden F, Birmann BM. Dietary fat intake and risk of non-Hodgkin lymphoma in 2 large prospective cohorts. American Journal of Clinical Nutrition. 2017;106(2):650-6. doi: 10.3945/ajcn.117.155010.

22.	Bianchi VE, Herrera PF, Laura R. Effect of nutrition on neurodegenerative diseases. A systematic review. Nutritional Neuroscience. 2021;24(10):810-34. doi: 10.1080/1028415X.2019.1681088.

23.	Boushey C, Ard J, Bazzano L, Heymsfield S, Mayer-Davis E, Sabaté J, et al. Dietary Patterns and Breast, Colorectal, Lung, and Prostate Cancer: A Systematic Review. Alexandria (VA): USDA Nutrition Evidence Systematic Review; 2020.

24.	Chai XN, Zhou BQ, Ning N, Pan T, Xu F, He SH, et al. Effects of lifestyle intervention on adults with metabolic associated fatty liver disease: A systematic review and meta-analysis. Frontiers in Endocrinology. 2023;14:1081096. doi: 10.3389/fendo.2023.1081096.

25.	Chawla S, Tessarolo Silva F, Amaral Medeiros S, Mekary RA, Radenkovic D. The Effect of Low-Fat and Low-Carbohydrate Diets on Weight Loss and Lipid Levels: A Systematic Review and Meta-Analysis. Nutrients. 2020;12(12):3774. doi: 10.3390/nu12123774.

26.	Da Costa Brum IS, Mafra D, Moreira LSG, Teixeira KTR, Stockler-Pinto MB, Cardozo LFMF, et al. Consumption of oils and anthocyanins may positively modulate PPAR-γ expression in chronic noncommunicable diseases: A systematic review. Nutrition Research. 2022;105:66-76. doi: 10.1016/j.nutres.2022.06.004.

27.	Dianatinasab M, Forozani E, Akbari A, Azmi N, Bastam D, Fararouei M, et al. Dietary patterns and risk of bladder cancer: a systematic review and meta-analysis. BMC Public Health. 2022;22(1):73. doi: 10.1186/s12889-022-12516-2.

28.	Dietary Guidelines Advisory Committee. Scientific Report of the 2020 Dietary Guidelines Advisory Committee: Advisory Report to the Secretary of Agriculture and the Secretary of Health and Human Services. Washington, DC: U.S. Department of Agriculture, Agricultural Research Service; 2020. https://doi.org/10.52570/DGAC2020.

29.	Filippou CD, Thomopoulos CG, Kouremeti MM, Sotiropoulou LI, Nihoyannopoulos PI, Tousoulis DM, et al. Mediterranean diet and blood pressure reduction in adults with and without hypertension: A systematic review and meta-analysis of randomized controlled trials. Clinical Nutrition. 2021;40(5):3191-200. doi: 10.1016/j.clnu.2021.01.030.

30.	Gabriel FC, Oliveira M, De Martino Martella B, Berk M, Brietzke E, Jacka FN, et al. Nutrition and bipolar disorder: a systematic review. Nutritional Neuroscience. 2023;26(7):637-51. doi: 10.1080/1028415X.2022.2077031.

31.	Garcêz LS, Avelar CR, Fonseca NSS, Costa PRF, Lyra AC, Cunha CM, et al. Effect of dietary carbohydrate and lipid modification on clinical and anthropometric parameters in nonalcoholic fatty liver disease: a systematic review and meta-analysis. Nutrition Reviews. 2021;79(12):1321-37. doi: 10.1093/nutrit/nuaa146.

32.	Gravesteijn E, Mensink RP, Plat J. Effects of nutritional interventions on BDNF concentrations in humans: a systematic review. Nutritional Neuroscience. 2022;25(7):1425-36. doi: 10.1080/1028415X.2020.1865758.

33.	Guerra BA, Pereira TG, Eckert IC, Bernardes S, Silva FM. Markers of respiratory function response to high-carbohydrate and high-fat intake in patients with lung diseases: a systematic review with meta-analysis of randomized clinical trials. Journal of Parenteral and Enteral Nutrition. 2022;46(7):1522-34. doi: 10.1002/jpen.2385.

34.	Gupta M, Liti B, Barrett C, Thompson PD, Fernandez AB. Prevention and Management of Hypertriglyceridemia-Induced Acute Pancreatitis During Pregnancy: A Systematic Review. The American Journal of Medicine. 2022;135(6):709-14. doi: 10.1016/j.amjmed.2021.12.006.

35.	Hooper L, Summerbell CD, Thompson R, Sills D, Roberts FG, Moore HJ, et al. Reduced or modified dietary fat for preventing cardiovascular disease. Sao Paulo Medical Journal. 2016;134(2):182-3. doi: 10.1590/1516-3180.20161342T1.

36.	Jaramillo AP, Abaza A, Sid Idris F, Anis H, Vahora I, Moparthi KP, et al. Diet as an Optional Treatment in Adults With Inflammatory Bowel Disease: A Systematic Review of the Literature. Cureus. 2023;15(7):e42057. doi: 10.7759/cureus.42057.

37.	Kaviyarasan S, Chung Sia EL, Retinasamy T, Arulsamy A, Shaikh MF. Regulation of gut microbiome by ketogenic diet in neurodegenerative diseases: A molecular crosstalk. Frontiers in Aging Neuroscience. 2022;14:1015837. doi: 10.3389/fnagi.2022.1015837.

38.	Khodarahmi M, Azadbakht L. Dietary fat intake and functional dyspepsia. Advanced Biomedical Research. 2016;5:76. doi: 10.4103/2277-9175.180988.

39.	Kiesswetter E, Stadelmaier J, Petropoulou M, Morze J, Grummich K, Roux I, et al. Effects of Dairy Intake on Markers of Cardiometabolic Health in Adults: A Systematic Review with Network Meta-Analysis. Advances in Nutrition. 2023;14(3):438-50. doi: 10.1016/j.advnut.2023.03.004.

40.	Lei L, Huang J, Zhang L, Hong Y, Hui S, Yang J. Effects of low-carbohydrate diets versus low-fat diets on metabolic risk factors in overweight and obese adults: A meta-analysis of randomized controlled trials. Frontiers in Nutrition. 2022;9:935234. doi: 10.3389/fnut.2022.935234.

41.	Li S, Ding L, Xiao X. Comparing the Efficacy and Safety of Low-Carbohydrate Diets with Low-Fat Diets for Type 2 Diabetes Mellitus Patients: A Systematic Review and Meta-Analysis of Randomized Clinical Trials. International Journal of Endocrinology. 2021;2021(1):8521756. doi: 10.1155/2021/8521756.

42.	Mayer-Davis E, Leidy H, Mattes R, Naimi T, Novotny R, Schneeman B, et al. Beverage Consumption and Growth, Size, Body Composition, and Risk of Overweight and Obesity: A Systematic Review. Alexandria (VA): USDA Nutrition Evidence Systematic Review; 2020. https://www.ncbi.nlm.nih.gov/books/NBK579053/.

43.	Muth AK, Park SQ. The impact of dietary macronutrient intake on cognitive function and the brain. Clinical Nutrition. 2021;40(6):3999-4010. doi: 10.1016/j.clnu.2021.04.043.

44.	Nelson J, Sjöblom H, Gjertsson I, Ulven SM, Lindqvist HM, Bärebring L. Do Interventions with Diet or Dietary Supplements Reduce the Disease Activity Score in Rheumatoid Arthritis? A Systematic Review of Randomized Controlled Trials. Nutrients. 2020;12(10):2991. doi: 10.3390/nu12102991.

45.	Panagiotou E, Andreou E, Nicolaou SA. The Effect of Dietary Components of the Mediterranean Diet on Food Allergies: A Systematic Review. Nutrients. 2023;15(15):3295. doi: 10.3390/nu15153295.

46.	Prado MBJ, Pedro KM, Adiao KJB. Efficacy, safety and tolerability of high caloric diet in amyotrophic lateral sclerosis patients: A systematic review and meta-analysis. Revue Neurologique. 2023;179(9):1008-19. doi: 10.1016/j.neurol.2023.01.731.

47.	Rosqvist F, Rydell A, Iggman D. The Effects of Foods on Blood Lipids in Non-alcoholic Fatty Liver Disease (NAFLD)-A Systematic Review and Meta-Analysis. Frontiers in Nutrition. 2020;7:613221. doi: 10.3389/fnut.2020.613221.

48.	Sabo CM, Simiras C, Ismaiel A, Dumitrascu DL. Diet and Gut Inflammation: The Effect of Diet on Inflammatory Markers in Inflammatory Bowel Disease - A Scoping Review. Journal of Gastrointestinal and Liver Diseases. 2023;32(3):402-10. doi: 10.15403/jgld-5090.

49.	Sanlier N, Sabuncular G. Relationship between nutrition and sleep quality, focusing on the melatonin biosynthesis. Sleep and Biological Rhythms. 2020;18(2):89-99. doi: 10.1007/s41105-020-00256-y.

50.	Santos EJF, Farisogullari B, Dures E, Geenen R, Machado PM, EULAR taskforce on recommendations for the management of fatigue in people with inflammatory rheumatic diseases. Efficacy of non-pharmacological interventions: a systematic review informing the 2023 EULAR recommendations for the management of fatigue in people with inflammatory rheumatic and musculoskeletal diseases. RMD Open. 2023;9(3):e003350. doi: 10.1136/rmdopen-2023-003350.

51.	Schoeler M, Ellero-Simatos S, Birkner T, Mayneris-Perxachs J, Olsson L, Brolin H, et al. The interplay between dietary fatty acids and gut microbiota influences host metabolism and hepatic steatosis. Nature Communications. 2023;14(1):5329. doi: 10.1038/s41467-023-41074-3.

52.	Schwab U, Reynolds AN, Sallinen T, Rivellese AA, Risérus U. Dietary fat intakes and cardiovascular disease risk in adults with type 2 diabetes: a systematic review and meta-analysis. European Journal of Nutrition. 2021;60(6):3355-63. doi: 10.1007/s00394-021-02507-1.

53.	Sindler D, Kastovska B, Dostal T, Cipryan L, Elavsky S. The effects of carbohydrate-restricted diet on psychological outcomes: a systematic review of randomized controlled trials. Nutrition Reviews. 2024;82(2):228-39. doi: 10.1093/nutrit/nuad053.

54.	Tarabanis C, Long C, Scolaro B, Heffron SP. Reviewing the cardiovascular and other health effects of olive oil: Limitations and future directions of current supplement formulations. Nutrition, Metabolism & Cardiovascular Diseases. 2023;33(12):2326-33. doi: 10.1016/j.numecd.2023.08.014.

55.	Treadwell JR, Kessler SK, Wu M, Abend NS, Massey SL, Tsou AY. Pharmacologic and Dietary Treatments for Epilepsies in Children Aged 1-36 Months: A Systematic Review. Neurology. 2023;100(1):e16-27. doi: 10.1212/WNL.0000000000201026.

56.	Uhomoibhi TO, Okobi TJ, Okobi OE, Koko JO, Uhomoibhi O, Igbinosun OE, et al. High-Fat Diet as a Risk Factor for Breast Cancer: A Meta-Analysis. Cureus. 2022;14(12):e32309. doi: 10.7759/cureus.32309.

57.	Urzì AG, Tropea E, Gattuso G, Spoto G, Marsala G, Calina D, et al. Ketogenic Diet and Breast Cancer: Recent Findings and Therapeutic Approaches. Nutrients. 2023;15(20):4357. doi: 10.3390/nu15204357.

58.	Wang Y, Liu J, Compher C, Kral TVE. Associations between dietary intake, diet quality and depressive symptoms in youth: A systematic review of observational studies. Health Promotion Perspectives. 2022;12(3):249-65. doi: 10.34172/hpp.2022.32.

59.	Willems AEM, Sura-de Jong M, Van Beek AP, Nederhof E, Van Dijk G. Effects of macronutrient intake in obesity: a meta-analysis of low-carbohydrate and low-fat diets on markers of the metabolic syndrome. Nutrition Reviews. 2021;79(4):429-44. doi: 10.1093/nutrit/nuaa044.

60.	Xu X, Zhang F, Ren J, Zhang H, Jing C, Wei M, et al. Dietary intervention improves metabolic levels in patients with type 2 diabetes through the gut microbiota: a systematic review and meta-analysis. Frontiers in Nutrition. 2024;10:1243095. doi: 10.3389/fnut.2023.1243095.

61.	Xu Y, Lu B, Zhou Y, Ren S, Pang G, Deng A. Is dietary fat associated with the risk of age-related macular degeneration? Protocol for a systematic review and meta-analysis. Medicine. 2020;99(17):e19081. doi: 10.1097/MD.0000000000019081.

62.	Yang JJ, Yu D, Takata Y, Smith-Warner SA, Blot W, White E, et al. Dietary Fat Intake and Lung Cancer Risk: A Pooled Analysis. Journal of Clinical Oncology. 2017;35(26):3055-64. doi: 10.1200/JCO.2017.73.3329.

63.	Yang Q, Lang X, Li W, Liang Y. The effects of low-fat, high-carbohydrate diets vs. low-carbohydrate, high-fat diets on weight, blood pressure, serum liquids and blood glucose: a systematic review and meta-analysis. European Journal of Clinical Nutrition. 2022;76(1):16-27. doi: 10.1038/s41430-021-00927-0.

64.	Yao Y, Tan P, Kim JE. Effects of dietary fats on the bioaccessibility and bioavailability of carotenoids: a systematic review and meta-analysis of in vitro studies and randomized controlled trials. Nutrition Reviews. 2022;80(4):741-61. doi: 10.1093/nutrit/nuab098.

65.	Zhao Q, Khedkar SV, Johnson KC. Weight Loss Interventions and Skeletal Health in Persons with Diabetes. Current Osteoporosis Reports. 2022;20(5):240-8. doi: 10.1007/s11914-022-00744-9.

66.	Becerra-Tomás N, Balducci K, Abar L, Aune D, Cariolou M, Greenwood DC, et al. Postdiagnosis dietary factors, supplement use and breast cancer prognosis: Global Cancer Update Programme (CUP Global) systematic literature review and meta-analysis. International Journal of Cancer. 2023;152(4):616-34. doi: 10.1002/ijc.34321.

67.	Brayner B, Kaur G, Keske MA, Marchese LE, Livingstone KM. Novel approach to investigate the association between type 2 diabetes risk and dietary fats in a dietary pattern context: a scoping review. Frontiers in Nutrition. 2023;10:1071855. doi: 10.3389/fnut.2023.1071855.

68.	Crane TE, Khulpateea BR, Alberts DS, Basen-Engquist K, Thomson CA. Dietary intake and ovarian cancer risk: a systematic review. Cancer Epidemiology, Biomarkers & Prevention. 2014;23(2):255-73. doi: 10.1158/1055-9965.EPI-13-0515.

69.	Del Bo C, Perna S, Allehdan S, Rafique A, Saad S, AlGhareeb F, et al. Does the Mediterranean Diet Have Any Effect on Lipid Profile, Central Obesity and Liver Enzymes in Non-Alcoholic Fatty Liver Disease (NAFLD) Subjects? A Systematic Review and Meta-Analysis of Randomized Control Trials. Nutrients. 2023;15(10) doi: 10.3390/nu15102250.

70.	Dietary Guidelines Advisory Committee Dietary Patterns Subcommittee. Dietary Patterns and Risk of Cardiovascular Disease: A Systematic Review. Alexandria, VA: U.S. Department of Agriculture, Food and Nutrition Service, Center for Nutrition Policy and Promotion; 2020 July 15. https://nesr.usda.gov/2020-dietary-guidelines-advisory-committee-systematic-reviews.

71.	El Sherbiny S, Squillacioti G, Colombi N, Ghelli F, Lenta E, Dalla Costa C, et al. The Effect of Dietary Patterns and Nutrient Intake on Oxidative Stress Levels in Pregnant Women: A Systematic Review. Antioxidants. 2023;12(7) doi: 10.3390/antiox12071427.

72.	Grisotto G, Farago JS, Taneri PE, Wehrli F, Roa-Díaz ZM, Minder B, et al. Dietary factors and onset of natural menopause: A systematic review and meta-analysis. Maturitas. 2022;159:15-32. doi: 10.1016/j.maturitas.2021.12.008.

73.	Heidarzadeh-Esfahani N, Soleimani D, Hajiahmadi S, Moradi S, Heidarzadeh N, Nachvak SM. Dietary intake in relation to the risk of reflux disease: a systematic review. Preventive Nutrition and Food Science. 2021;26(4):367-79. doi: 10.3746/pnf.2021.26.4.367.

74.	Leed A, Sheridan E, Baker B, Bamford S, Emmanouilidis E, Stewart F, et al. Dietary Intake and Arterial Stiffness in Children and Adolescents: A Systematic Review. Nutrients. 2023;15(9) doi: 10.3390/nu15092092.

75.	Liang S, Mijatovic J, Li A, Koemel N, Nasir R, Toniutti C, et al. Dietary patterns and non-communicable disease biomarkers: a network meta-analysis and nutritional geometry approach. Nutrients. 2022;15(1):76. doi: 10.3390/nu15010076.

76.	Luong R, Ribeiro RV, Cunningham J, Chen S, Hirani V. The short-and long-term effects of dietary patterns on cardiometabolic health in adults aged 65 years or older: a systematic review. Nutrition Reviews. 2022;80(3):329-50. doi: 10.1093/nutrit/nuab032.

77.	Ma Y, Zheng Z, Zhuang L, Wang H, Li A, Chen L, et al. Dietary Macronutrient Intake and Cardiovascular Disease Risk and Mortality: A Systematic Review and Dose-Response Meta-Analysis of Prospective Cohort Studies. Nutrients. 2024;16(1) doi: 10.3390/nu16010152.

78.	Mourouti N, Kontogianni MD, Papavagelis C, Panagiotakos DB. Diet and breast cancer: a systematic review. International Journal of Food Sciences and Nutrition. 2015;66(1):1-42. doi: 10.3109/09637486.2014.950207.

79.	Snetselaar LG, Cheek JJ, Fox SS, Healy HS, Schweizer ML, Bao W, et al. Efficacy of diet on fatigue and quality of life in multiple sclerosis: a systematic review and network meta-analysis of randomized trials. Neurology. 2023;100(4):e357-e66. doi: 10.1212/WNL.0000000000201371.

80.	Townsend RF, Logan D, O’Neill RF, Prinelli F, Woodside JV, McEvoy CT. Whole dietary patterns, cognitive decline and cognitive disorders: a systematic review of prospective and intervention studies. Nutrients. 2023;15(2):333. doi: 10.3390/nu15020333.

81.	Wang A, Lin Y, Wu Y, Zhang D. Macronutrients intake and risk of Parkinson's disease: A meta-analysis. Geriatrics & Gerontology International. 2015;15(5):606-16. doi: 10.1111/ggi.12321.

82.	Winters-van Eekelen E, Verkouter I, Peters HP, Alssema M, de Roos BG, Schrauwen-Hinderling VB, et al. Effects of dietary macronutrients on liver fat content in adults: a systematic review and meta-analysis of randomized controlled trials. European Journal of Clinical Nutrition. 2021;75(4):588-601. doi: 10.1038/s41430-020-00778-1.

83.	Wu Y-C, Zheng D, Sun J-J, Zou Z-K, Ma Z-L. Meta-analysis of studies on breast cancer risk and diet in Chinese women. International Journal of Clinical and Experimental Medicine. 2015;8(1):73-85. doi: NONE.

84.	Zeng L, Hu S, Chen P, Wei W, Tan Y. Macronutrient Intake and Risk of Crohn's Disease: Systematic Review and Dose-Response Meta-Analysis of Epidemiological Studies. Nutrients. 2017;9(5) doi: 10.3390/nu9050500.

85.	Zhang M, Hou Z-K, Huang Z-B, Chen X-L, Liu F-B. Dietary and lifestyle factors related to gastroesophageal reflux disease: a systematic review. Therapeutics and Clinical Risk Management. 2021;17:305-23. doi: 10.2147/TCRM.S296680.

86.	Cao Y, Hou L, Wang W. Dietary total fat and fatty acids intake, serum fatty acids and risk of breast cancer: A meta-analysis of prospective cohort studies. International Journal of Cancer. 2016;138(8):1894-904. doi: 10.1002/ijc.29938.

87.	Julibert A, Bibiloni MDM, Tur JA. Dietary fat intake and metabolic syndrome in adults: A systematic review. Nutrition, Metabolism & Cardiovascular Diseases. 2019;29(9):887-905. doi: 10.1016/j.numecd.2019.05.055.

88.	Kim Y, Je Y, Giovannucci EL. Association between dietary fat intake and mortality from all-causes, cardiovascular disease, and cancer: A systematic review and meta-analysis of prospective cohort studies. Clinical Nutrition. 2021;40(3):1060-70. doi: 10.1016/j.clnu.2020.07.007.

89.	Mazidi M, Mikhailidis DP, Sattar N, Toth PP, Judd S, Blaha MJ, et al. Association of types of dietary fats and all-cause and cause-specific mortality: A prospective cohort study and meta-analysis of prospective studies with 1,164,029 participants. Clinical Nutrition. 2020 doi: 10.1016/j.clnu.2020.03.028.

90.	Ruan L, Cheng S-P, Zhu Q-X. Dietary fat intake and the risk of skin cancer: a systematic review and meta-analysis of observational studies. Nutrition and Cancer. 2020;72(3):398-408. doi: 10.1080/01635581.2019.1637910.

91.	Sadeghi A, Shab-Bidar S, Parohan M, Djafarian K. Dietary Fat Intake and Risk of Ovarian Cancer: A Systematic Review and Dose-Response Meta-Analysis of Observational Studies. Nutrition and Cancer. 2019;71(6):939-53. doi: 10.1080/01635581.2019.1595049.

92.	Schwab U, Lauritzen L, Tholstrup T, Haldorssoni T, Riserus U, Uusitupa M, et al. Effect of the amount and type of dietary fat on cardiometabolic risk factors and risk of developing type 2 diabetes, cardiovascular diseases, and cancer: a systematic review. Food & Nutrition Research. 2014;58 doi: 10.3402/fnr.v58.25145.

93.	Visser E, Ten Brinke A, Sizoo D, Pepels JJS, Ten Have L, van der Wiel E, et al. Effect of dietary interventions on markers of type 2 inflammation in asthma: A systematic review. Respiratory medicine. 2024;221:107504. doi: 10.1016/j.rmed.2023.107504.

94.	Von Frankenberg AD, Silva FM, de Almeida JC, Piccoli V, do Nascimento FV, Sost MM, et al. Effect of dietary lipids on circulating adiponectin: a systematic review with meta-analysis of randomised controlled trials. British Journal of Nutrition. 2014;112(8):1235-50. doi: 10.1017/S0007114514002013.

95.	Wang F, Lin X, Zhao Q, Li J. Fat intake and risk of ulcerative colitis: Systematic review and dose-response meta-analysis of epidemiological studies. Journal of Gastroenterology and Hepatology. 2017;32(1):19-27. doi: 10.1111/jgh.13416.

96.	Wang J, Wang C. Dietary fat intake and risk of bladder cancer: Evidence from a meta-analysis of observational studies. Cellular and Molecular Biology. 2019;65(7):5-9. doi: 10.14715/cmb/2019.65.7.2.

97.	Zhao L, Deng C, Lin Z, Giovannucci E, Zhang X. Dietary fats, serum cholesterol and liver cancer risk: a systematic review and meta-analysis of prospective studies. Cancers. 2021;13(7):1580. doi: 10.3390/cancers13071580.

98.	Zhu RZ, Chen MQ, Zhang ZW, Wu TY, Zhao WH. Dietary fatty acids and risk for Alzheimer's disease, dementia, and mild cognitive impairment: A prospective cohort meta-analysis. Nutrition 2021;90:111355. doi: 10.1016/j.nut.2021.111355.

99.	Zhu Y, Bo Y, Liu Y. Dietary total fat, fatty acids intake, and risk of cardiovascular disease: a dose-response meta-analysis of cohort studies. Lipids in Health and Disease. 2019;18(1):91. doi: 10.1186/s12944-019-1035-2.

100.	Jayedi A, Soltani S, Emadi A, Ghods K, Shab-Bidar S. Dietary intake, biomarkers and supplementation of fatty acids and risk of coronary events: a systematic review and dose-response meta-analysis of randomized controlled trials and prospective observational studies. Critical reviews in food science and nutrition. 2023:1-20. doi: 10.1080/10408398.2023.2251583.

101.	Karam G, Agarwal A, Sadeghirad B, Jalink M, Hitchcock CL, Ge L, et al. Comparison of seven popular structured dietary programmes and risk of mortality and major cardiovascular events in patients at increased cardiovascular risk: systematic review and network meta-analysis. BMJ (Clinical research ed). 2023;380:e072003. doi: 10.1136/bmj-2022-072003.

102.	Katsagoni CN, Papachristou E, Sidossis A, Sidossis L. Effects of dietary and lifestyle interventions on liver, clinical and metabolic parameters in children and adolescents with non-alcoholic fatty liver disease: a systematic review. Nutrients. 2020;12(9):2864. doi: 10.3390/nu12092864.

103.	Shen QW, Yao QY. Total fat consumption and pancreatic cancer risk: a meta-analysis of epidemiologic studies. European Journal of Cancer Prevention. 2015;24(4):278-85. doi: 10.1097/CEJ.0000000000000073.

104.	Han TJ, Li JS, Luan XT, Wang L, Xu HZ. Dietary Fat Consumption and Non-Hodgkin's Lymphoma Risk: A Meta-analysis. Nutrition and Cancer. 2017;69(2):221-8. doi: 10.1080/01635581.2017.1263753.

105.	Mozaffari H, Djafarian K, Mofrad MD, Shab-Bidar S. Dietary fat, saturated fatty acid, and monounsaturated fatty acid intakes and risk of bone fracture: a systematic review and meta-analysis of observational studies. Osteoporosis International. 2018;29(9):1949-61. doi: 10.1007/s00198-018-4540-7.

106.	Lu M, Wan Y, Yang B, Huggins CE, Li D. Effects of low-fat compared with high-fat diet on cardiometabolic indicators in people with overweight and obesity without overt metabolic disturbance: a systematic review and meta-analysis of randomised controlled trials. British Journal of Nutrition. 2018;119(1):96-108. doi: 10.1017/S0007114517002902.

107.	Schwingshackl L, Hoffmann G. Comparison of the long-term effects of high-fat v. low-fat diet consumption on cardiometabolic risk factors in subjects with abnormal glucose metabolism: a systematic review and meta-analysis. British Journal of Nutrition. 2014;111(12):2047-58. doi: 10.1017/S0007114514000464.

108.	Ruan Y, Tang J, Guo X, Li K, Li D. Dietary Fat Intake and Risk of Alzheimer's Disease and Dementia: A Meta-Analysis of Cohort Studies. Current Alzheimer Research. 2018;15(9):869-76. doi: 10.2174/1567205015666180427142350.

109.	Wu J, Xue P, Yang C, He H, Mao B. Dietary fat and risk of inflammatory bowel disease: a meta-analysis. International Journal of Clinical and Experimental Medicine. 2016;9(6):11072-7. doi: NONE.

110.	Whittaker J, Wu K. Low-fat diets and testosterone in men: Systematic review and meta-analysis of intervention studies. The Journal of Steroid Biochemistry and Molecular Biology. 2021;210:105878. doi: 10.1016/j.jsbmb.2021.105878.

111.	Gopinath A, Cheema AH, Chaludiya K, Khalid M, Nwosu M, Agyeman WY, et al. The impact of dietary fat on breast cancer incidence and survival: a systematic review. Cureus. 2022;14(10) doi: 10.7759/cureus.30003.

112.	Bu Y, Qu J, Ji S, Zhou J, Xue M, Qu J, et al. Dietary patterns and breast cancer risk, prognosis, and quality of life: A systematic review. Frontiers in Nutrition. 2023;9:1057057. doi: 10.3389/fnut.2022.1057057.

113.	Han J, Jiang Y, Liu X, Meng Q, Xi Q, Zhuang Q, et al. Dietary fat intake and risk of gastric cancer: a meta-analysis of observational studies. PLoS One. 2015;10(9):e0138580. doi: 10.1371/journal.pone.0138580.

114.	He D, Huang X, Wang Z-P, Chen D, Chen J, Duan C-Y. Dietary fat intake and risk of esophageal carcinoma: a meta-analysis of observational studies. Oncotarget. 2017;8(58):99049. doi: 10.18632/oncotarget.21462.

115.	Kim M, Park K. Dietary fat intake and risk of colorectal cancer: a systematic review and meta-analysis of prospective studies. Nutrients. 2018;10(12):1963. doi: 10.3390/nu10121963.

116.	Vahid F, Davoodi SH. Nutritional factors involved in the etiology of gastric cancer: a systematic review. Nutrition and Cancer. 2021;73(3):376-90. doi: 10.1080/01635581.2020.1756353.

117.	Hou R, Wu Q-J, Gong T-T, Jiang L. Dietary fat and fatty acid intake and epithelial ovarian cancer risk: evidence from epidemiological studies. Oncotarget. 2015;6(40):43099. doi: 10.18632/oncotarget.5525.

118.	Qiu W, Lu H, Qi Y, Wang X. Dietary fat intake and ovarian cancer risk: a meta-analysis of epidemiological studies. Oncotarget. 2016;7(24):37390-406. doi: 10.18632/oncotarget.8940.

119.	Zhang X, Ding HM, Deng LF, Chen GC, Li J, He ZY, et al. Dietary fats and serum lipids in relation to the risk of ovarian cancer: a meta-analysis of observational studies. Front Nutr. 2023;10:1153986. doi: 10.3389/fnut.2023.1153986.

120.	Zhao J, Lyu C, Gao J, Du L, Shan B, Zhang H, et al. Dietary fat intake and endometrial cancer risk: A dose response meta-analysis. Medicine. 2016;95(27):e4121. doi: 10.1097/MD.0000000000004121.

121.	Xu C, Han FF, Zeng XT, Liu TZ, Li S, Gao ZY. Fat Intake Is Not Linked to Prostate Cancer: A Systematic Review and Dose-Response Meta-Analysis. PLoS One. 2015;10(7):e0131747. doi: 10.1371/journal.pone.0131747.

122.	Rinninella E, Mele MC, Cintoni M, Raoul P, Ianiro G, Salerno L, et al. The facts about food after cancer diagnosis: a systematic review of prospective cohort studies. Nutrients. 2020;12(8):2345. doi: 10.3390/nu12082345.

123.	Barkas F, Nomikos T, Liberopoulos E, Panagiotakos D. Diet and cardiovascular disease risk among individuals with familial hypercholesterolemia: systematic review and meta-analysis. Nutrients. 2020;12(8):2436. doi: 10.3390/nu12082436.

124.	Kim J, Hoang T, Bu SY, Kim J-M, Choi J-H, Park E, et al. Associations of dietary intake with cardiovascular disease, blood pressure, and lipid profile in the Korean population: a systematic review and meta-analysis. Journal of Lipid and Atherosclerosis. 2020;9(1):205-29. doi: 10.12997/jla.2020.9.1.205.

125.	Keshani M, Feizi A, Askari G, Sharma M, Bagherniya M. Effects of therapeutic lifestyle change diets on blood lipids, lipoproteins, glycemic parameters, and blood pressure: a systematic review and meta-analysis of clinical trials. Nutrition Reviews. 2024;82(2):176-92. doi: 10.1093/nutrit/nuad051.

126.	Jing T, Zhang S, Bai M, Chen Z, Gao S, Li S, et al. Effect of Dietary Approaches on Glycemic Control in Patients with Type 2 Diabetes: A Systematic Review with Network Meta-Analysis of Randomized Trials. Nutrients. 2023;15(14) doi: 10.3390/nu15143156.

127.	Swedish Agency for Health Technology Assessment and Assessment of Social Services (SBU). Diets for diabetes: A systematic review and assessment of effects, health economic and ethical aspects. Stockholm, Sweden: Swedish Agency for Health Technology Assessment and Assessment of Social Services (SBU); 2022.

128.	Attuquayefio T, Stevenson RJ. A systematic review of longer-term dietary interventions on human cognitive function: Emerging patterns and future directions. Appetite. 2015;95:554-70. doi: 10.1016/j.appet.2015.08.023.

129.	Cao GY, Li M, Han L, Tayie F, Yao SS, Huang Z, et al. Dietary Fat Intake and Cognitive Function among Older Populations: A Systematic Review and Meta-Analysis. The Journal of Prevention of Alzheimer's Disease. 2019;6(3):204-11. doi: 10.14283/jpad.2019.9.

130.	Qu Y, Chen X, Xu MM, Sun Q. Relationship between high dietary fat intake and Parkinson's disease risk: a meta-analysis. Neural Regeneration Research. 2019;14(12):2156-63. doi: 10.4103/1673-5374.262599.

131.	Frediani JK, Lal AA, Kim E, Leslie SL, Boorman DW, Singh V. The role of diet and non-pharmacologic supplements in the treatment of chronic neuropathic pain: A systematic review. Pain Practice. 2024;24(1):186-210. doi: 10.1111/papr.13291.

132.	Varkaneh HK, Poursoleiman F, Al Masri MK, Alras KA, Shayah Y, Masmoum MD, et al. Low fat diet versus low carbohydrate diet for management of non-alcohol fatty liver disease: A systematic review. Frontiers in Nutrition. 2022;9:987921. doi: 10.3389/fnut.2022.987921.
